# SSR4 Sustains Tertiary Lymphoid Structures by Regulation Quality Control of N‐linked Glycosylation During B‐cell Differentiation Into Plasmacyte in Colorectal Cancer

**DOI:** 10.1002/advs.75790

**Published:** 2026-05-20

**Authors:** Wei Zhao, Youcai Zhao, Haiyang Li, Bowen Yang, Siyan Lu, Xueting Fu, Caixia Zhang, Yirong Chen, Xiaofeng Bian, Xinghua Ma, Xiaobing Yang, Bin Wang, Yuan Fang, Lulu Yang, Yelin Zhao, Shuli Zhao

**Affiliations:** ^1^ Department of Pathology Nanjing First Hospital Nanjing Medical University Nanjing Jiangsu China; ^2^ Department of General Surgery Nanjing First Hospital Nanjing Medical University Nanjing Jiangsu China; ^3^ General Clinical Research Center Nanjing First Hospital China Pharmaceutical University Nanjing Jiangsu China; ^4^ General Clinical Research Center Nanjing First Hospital Nanjing Medical University Nanjing Jiangsu China; ^5^ Division of ENT Diseases Department of Clinical Science Intervention and Technology (CLINTEC) Karolinska Institutet Stockholm Sweden

**Keywords:** B‐cell, colorectal cancer, N‐linked glycosylation, SSR4, tertiary lymphoid structures, tumor‐infiltrating B cells (TIBs)

## Abstract

Intratumoral tertiary lymphoid structure (TLS) fosters B‐cell differentiation and antibody production, yet the intrinsic programs sustaining these functions remain unclear. By integrating spatial transcriptomics and single‐cell RNA sequencing of colorectal cancer, we delineate a B‐cell trajectory enriched for endoplasmic reticulum protein processing, autophagy, NF‐κB signaling, and N‐linked glycosylation, highlighting SSR4 as a progressively induced TRAP‐complex component. SSR4 is further upregulated in B cells from anti‐PD‐1 responders. B‐cell‐specific *Ssr4* deletion leads to peripheral B‐cell loss, reduced antibody output, exacerbated colitis, and impaired mucosal immunity. Mechanistically, SSR4 interacts with DDOST to regulate BAFFR N‐glycosylation, thereby sustaining B‐cell activation and LTα1β2 expression via NF‐κB signaling. In *Apc* ^Min/+^ mice, *Ssr4* deficiency accelerates tumorigenesis, disrupts TLS maturation, lowers serum IgG1, and increases high‐mannose (HM) Ig. Conversely, SSR4 overexpression in CHO cells reduces high‐mannose glycans and enhances IgG1 ADCC and CDC. These findings identify SSR4 as a central regulator of B‐cell glycosylation and TLS‐dependent anti‐tumor immunity, offering translational implications for immunotherapy and antibody engineering.

## Graphical abstract

SSR4, a TRAP component induced in B cells, governs BAFFR N‐glycosylation via DDOST to sustain NF‐κB signaling, B‐cell differentiation, and TLS maturation. Its loss impairs anti‐tumor immunity, while overexpression improves antibody glycosylation and ADCC, revealing a critical regulator for cancer immunotherapy.

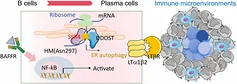

## 1 Introduction

Although the role of tumor‐infiltrating B cells (TIBs) in anti‐tumor immunity remains controversial [1], the presence of B cells and mature tertiary lymphoid structure (TLS) predicts therapeutic responses to immunotherapy and survival, more accurately than T cells in some solid tumors [2]. Tumor‐infiltrating B cells display multiple functions, primarily through their ability to differentiate into plasma cells to produce antibodies directed toward tumor‐associated antigens in some cancers [3, 4], so exploring the regulatory factors affecting B cell differentiation and development taking place inside tumors, particularly at the TLS site, is expected to improve the response to immunotherapy.

Nascent immunoglobulin (Ig) heavy and light chains are co‐translationally translocated into the lumen of the endoplasmic reticulum (ER), where Ig chains fold and assemble into functional, antigen‐binding H2L2 molecules [5, 6]. Glycosylation is a common and important post‐translational modification. The glycans on proteins are usually synthesized in ER first and then are translocated to the Golgi apparatus to be further modified by several resident glycosidases and glycosyltranferases to form hybrid‐type or complex‐type glycans [7]. Therefore, correct glycosylation modification is a key factor for the precise folding and conformation of Ig in the lumen of the endoplasm reticulum, which helps to improve the solubility, stability, and transport capacity of proteins, while those misfolded proteins enter the calreticulin/calnex cycle and are degraded by the autophagy pathway of ER‐associated degradation.

Protein glycosylation is a constitutive biosynthetic process coordinated by essential enzymes and is actively regulated by specific molecular mechanisms to ensure glycosylation under diverse cellular conditions [8]. The oligosaccharyltransferase (OST) complex, a polymeric protein complex consisting of the STT3A and STT3B catalytic subunits and a series of auxiliary proteins (RPN1, RPN2, TMEM258, DAD1, OST4, DDOST), is crucial for ensuring the correct glycosylation of proteins and is involved in transferring oligosaccharide chains onto proteins within the ER [9]. Recent research showed that the OST is associated with the ER translocation machinery via the heterotetrameric translocon‐associated protein (TRAP) complex (SSR1, SSR2, SSR3, and SSR4), and TRAP is required for translation efficiency and maturation of secretory proteins [8, 10].

The Signal Sequence Receptor Subunit 4 (SSR4) gene, a member of TRAP family that is responsible for encoding the δ subunit of this protein complex, participates in the transmembrane transportation of the protein on the ER membrane [11, 12]. Recent studies have also found that the SSR4 gene deletion can lead to ly‐ and le‐type congenital glycosylation disorders (CGD); meanwhile overexpression of SSR4 partially restores N‐linker glycosylation function [12]. In addition, SSR4 was found to be predominantly expressed in B cells [13]. However, the molecular mechanism of how it affects B cell differentiation and infiltration by regulating glycosylation modification has not been reported yet.

Here, by employing B cell‐conditional *Ssr4*‐knockout mice (*Ssr4* ^BKO^), we identified that SSR4 functions as a regulator on B cell development and immunoglobulin production under homeostatic conditions as well as T cell‐dependent (TD) and T cell‐independent (TI) antigenic stimulation. SSR4 deficiency also impairs B cell activation by disrupting NF‐κB signaling pathway. Mechanistically, SSR4 binding to N‐glycantransferase subunit DDOST to modify the N‐linked glycosylation of BAFFR on B cells. Moreover, the results from the enteritis and spontaneous intestinal adenomas animal models showed that SSR4 deficiency reshapes the tumor immune microenvironment (TIM) by affecting the migration and aggregation of B cells, the formation of tertiary lymphoid structure (TLS), and interfering with immunoglobulin glycosylation and immunological effects.

## 2 Results

### 2.1 In Situ Identification of CD19^+^ B‐cell‐enriched Spots in CRC Using Spatial Transcriptomic Data

**FIGURE 1:**
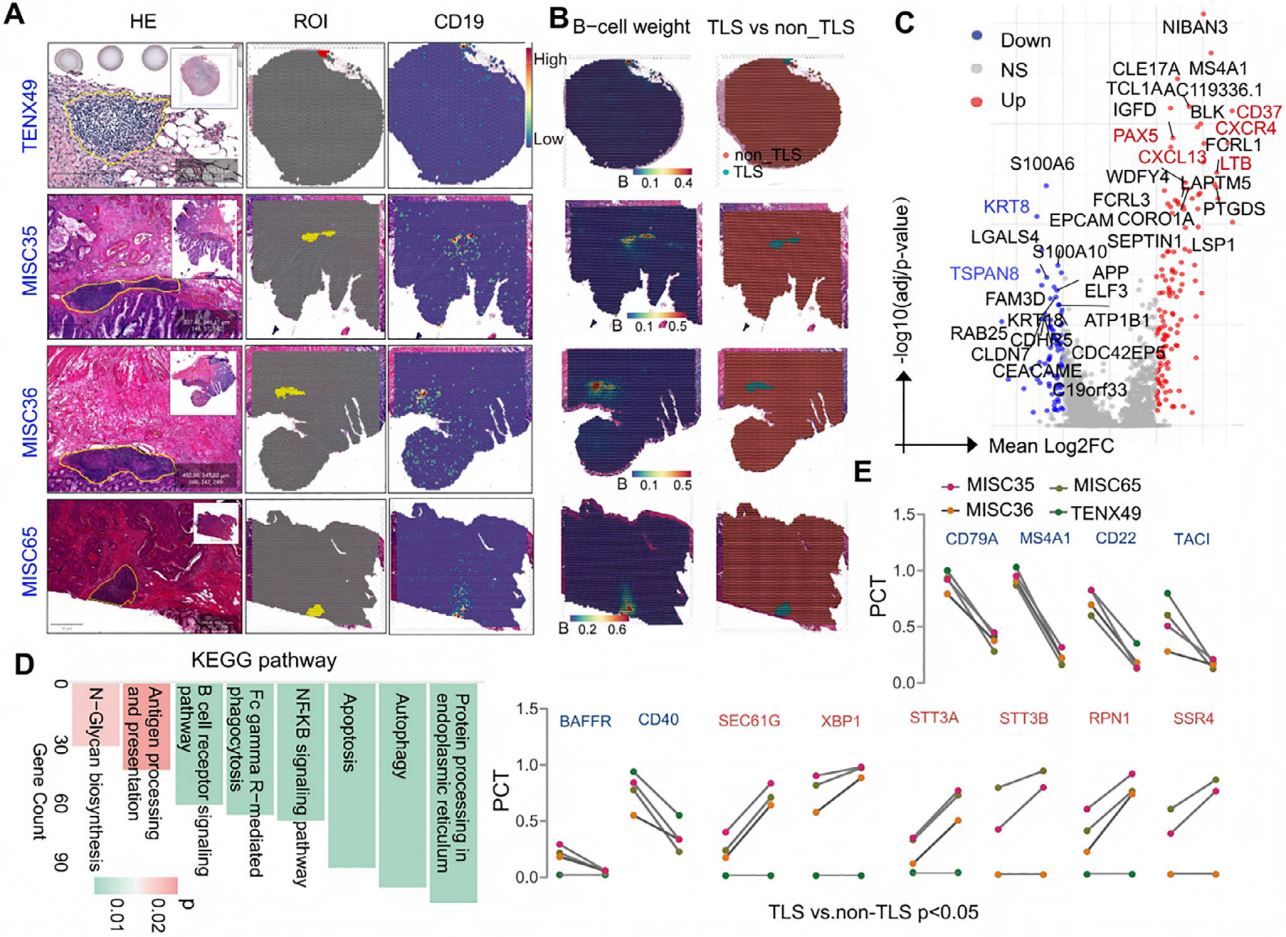
Spatial transcriptomic profiling reveals distinct transcriptional programs of B cells within tertiary lymphoid structures (TLS) in colorectal cancer (CRC). (A) Workflow for TLS identification and B cell characterization in CRC tissues using spatial transcriptomics (ST). TLS regions were defined through a dual approach combining manual pathological annotation and TLS‐specific gene signature scoring (n = 4). (B) ST visualization of B cell weight per spot, quantified by Robust Cell Type Decomposition (RCTD) deconvolution. (C) Differentially expressed genes between CD19^+^ B cells located in‐TLS and those in non‐TLS tumor stroma. B cells within TLS exhibited upregulation of genes associated with B cell activation, migration, and lymphoid organization (CD37, CXCR4, LTB, CXCL13, PAX5) and downregulation of epithelial‐associated genes (KRT8, TSPAN8). (D) KEGG pathway enrichment analysis showing that TLS‐associated B cells were enriched in signaling pathways related to protein processing in the endoplasmic reticulum, autophagy, NF‐κB signaling, and N‐glycan biosynthesis, suggesting that B cell functional heterogeneity may be linked to ER‐mediated protein transport and modification. Enrichment analysis was performed using the clusterProfiler R package; statistical significance was defined as adjusted *p* < 0.05. (E) The expression differences of key genes in TLS vs non‐TLS. For (C) and (E), differential expression analysis was performed using the two‐sided Wilcoxon rank‐sum test (Seurat package) with Benjamini–Hochberg FDR correction; data are presented as gene expression levels; statistical significance was defined as adjusted *p* < 0.05.

Although B cells correlate with improved survival in several solid tumors, including CRC, heterogeneity of B cell function in the tumor microenvironment (TME) complicates their identification using traditional marker panels. In view of tertiary lymphoid structures (TLS) in TME, which can support the development of B cell function, we employed spatial transcriptomic data from 4 CRC tissue samples containing TLS obtained from the HEST database to comprehensively profile B cell characteristics and their interplays with the TME (Table S1). To ensure comprehensive and biologically grounded TLS identification, we applied a dual method of manual annotation by pathologists and identification of 5 TLS‐specific signatures to quantify molecular features characteristic of TLS (Figure 1A,B and Figure S1A,B). To reveal the developmental trajectory and function of tumor‐infiltrating B cells in TME, we compared the differentially expressed genes between CD19^+^ B‐cell‐enriched spots in TLS and non‐TLS. Compared with those in non‐TLS, CD19^+^ B‐cell‐enriched spots in TLS had higher expression of genes associated with the function, development, migration of B lymphocytes, and the structure of lymphoid organs (CD37, CXCR4, LTB, CXCL13, PAX5), and lowly expressed KRT8, TSPAN8, and other genes (Figure 1C). And, the KEGG pathway analysis results showed that these differentially expressed genes were mainly enriched in signaling pathways such as protein processing in the endoplasmic reticulum (ER), autophagy, NF‐κB signaling pathway, and N‐glycan biosynthesis (Figure 1D). To further characterize the molecular features affecting antibody production, we examined the expression of key genes involved in the ER translocation machinery within CD19^+^ B‑cell‑enriched spots. Notably, components of the translocon‐associated protein (TRAP) subunit SSR4, protein translocation core channel SEC61G, oligosaccharyltransferase (OST) subunits (STT3A, STT3B, RPN1), and the unfolded protein response regulator XBP1 were consistently expressed at high levels in non‐TLS‑localized B‑cell spots, suggesting that the TLS microenvironment specifically affects the activation of ER‑associated protein processing machinery in infiltrating B cells (Figure 1E). These results suggested that tumor‐infiltrating B cells exhibit distinct transcriptional and functional programs in the CRC TME, and the functional heterogeneity of tumor‐infiltrating B cells is closely related to the TLS microenvironment, which may be mediated by the regulation of protein transport and modification in the ER of B cells.

### 2.2 Associations of SSR4 With the Differentiation of Tumor‐infiltrating B Cells in CRC

**FIGURE 2:**
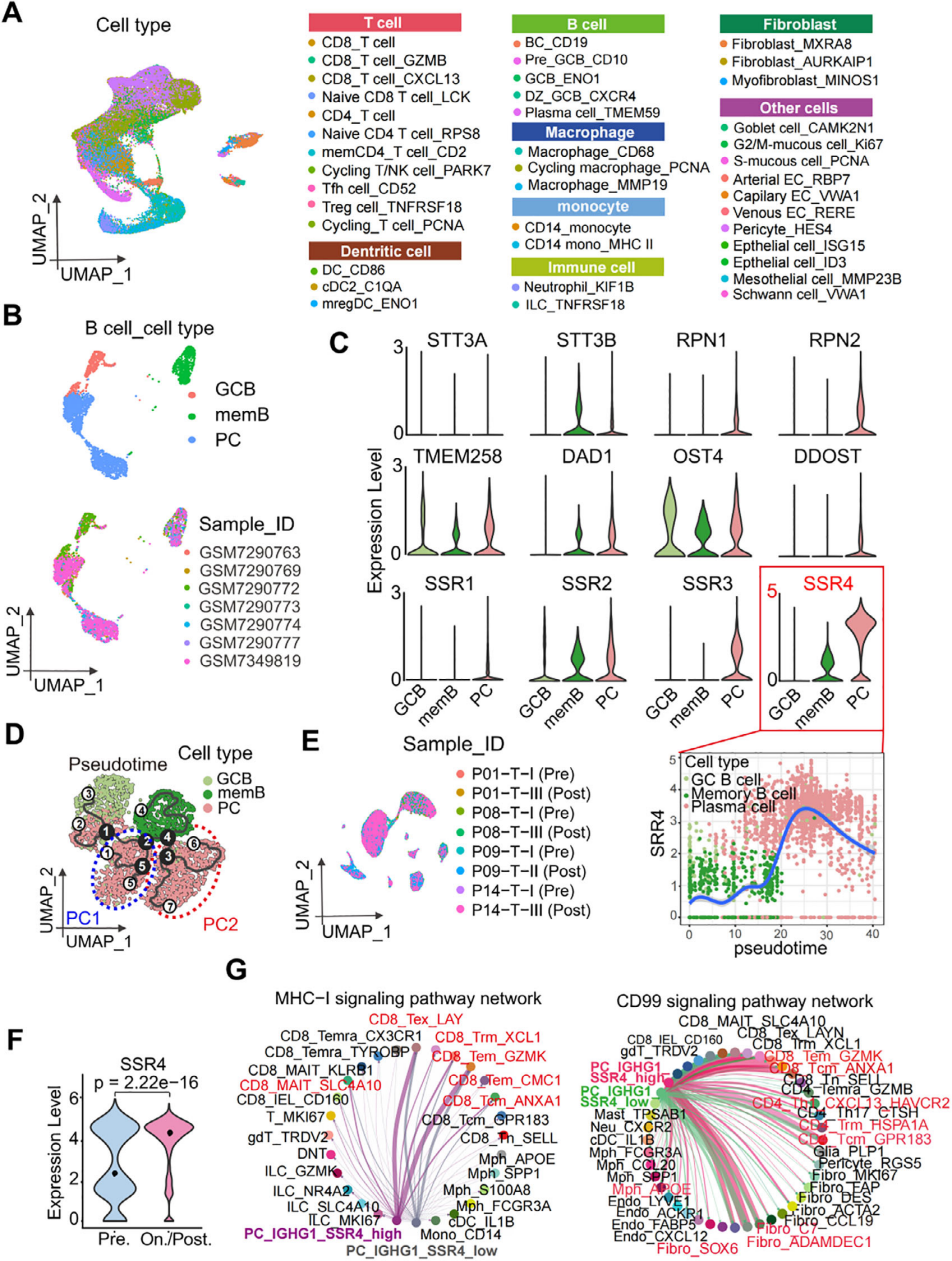
TRAP/OST complex‐mediated protein transport and glycosylation are linked to plasma cell differentiation and immunotherapy response in colorectal cancer (CRC). (A) Overview of the single‐cell transcriptomic analysis pipeline. Single‐cell RNA‐seq data from 7 CRC patients (41,667 high‐quality cells) were obtained from the DISCO database and classified into six major cell types, including T cell, Dentritic cell, B cell, Macrophage, Monocyte, Immune cell, Fibroblast, and Other cells, based on canonical marker expression. (B) Unsupervised clustering identified distinct B cell subsets representing different maturation stages and 7 CRC tissues. (C) Violin chart shows the differential expression of the TRAP complex genes (SSR1‐4) and OST complex genes (STT3A/B, RPN1/2, TMEM258, DAD1, OST4, DDOST) across B cell subsets; pseudotemporal differentiation map showing SSR4 expression dynamics in distinct B cell subtypes (down). (D) UMAP showing the result of pseudo‐time series analysis of B cell subtypes. (E) UMAP showing sample distribution of single cell transcriptome sequencing samples from 8 tissue samples of 4 additional CRC patients with paired pre‐ and post‐PD‐1 inhibitor treatment (GSE236581). (F) The violin diagram showing the changes of SSR4 expression in B cells at different treatment stages. Differential expression analysis was performed using the two‐sided Wilcoxon rank‐sum test (Seurat package) with Benjamini–Hochberg FDR correction; data are presented as violin plots (gene expression levels); statistical significance was defined as adjusted *p* < 0.05. (G) The network diagram illustrates the interaction patterns between IGHG1_PC with varying SSR4 expression levels and other cell types. Cell–cell interaction analysis was performed using CellChat; differences were assessed based on permutation tests within the package; statistical significance was defined as *p* < 0.05.

N‐glycosylation primarily facilitates the proper folding of nascent polypeptides, takes place in the ER lumen mediated by a membrane‐associated enzyme complex known as oligosaccharyltransferase (OST) [8] and the translocon‐associated protein (TRAP) complex [14]. To investigate the differences in gene expression related to protein transport (the TRAP complex genes, SSR1/SSR2/SSR3/SSR4) and glycosylation modification (OST complex genes, STT3A/ STT3B/ RPN1/ RPN2/ TMEM258/ DAD1/ OST4/ DDOST) in tumor‐infiltrating B cells, we analyzed single‐cell data from 7 colorectal cancers (CRC) patients obtained from Deeply Integrated human Single‐Cell Omics (DISCO) database (Figure S2A). The transcriptomes of 41,667 high‐quality single cells were obtained after stringent quality control (STAR Methods). Unsupervised clustering combined with canonical marker‐based annotation revealed six major cell types: T cell, Dentritic cell, B cell, Macrophage, Monocyte, Immune cell, Fibroblast, and Other cells (Figure 2A). Unsupervised clustering analysis identified typical B cell subsets representing different B cell maturation stages were identified, including germinal center (GC) B cell (CD19, RGS13, CD79A, and STMN1), memory B (memB) cells (CD19, TNFRSF13B, IGHM, and HLA‐DQB1), and plasma cells (CD19, MZB1, and JCHAIN) (Figure 2B). Of note, as previously reported by Ma et al. [15], tumor‐infiltrating plasma cells in CRC could be derived from canonical GCB and memB.

As an antibody‐secreting cell, the protein transport and glycosylation modification play a vital role in the fate and function of of plasma cells. Among the above 12 genes, SSR4 has the highest expression in plasma cells and shows significant expression in both two differentiation sources of plasma cells. Of the above 12 genes, the expression level of SSR4 was the highest in plasma cells derived from GCB and memB (Figure 2C and Figure S2C).

By comparing the differential gene expression profiles of plasma cells (PC1, derived from GCB, PC2 derived from memB), we found that ribosome‐related functional genes, such as cytoplasmic translation, ribonucleoprotein biogenesis, and rRNA metabolic, were significantly upregulated in PC1 (Figure 2D and Figure S2D), suggesting that tumor‐infiltrating plasma cells from GCB cells have a stronger antibody‐producing capacity [16].

To explore the relationship between SSR4 expression in tumor‐infiltrating plasma cells and immunotherapy outcomes in CRC patients, we analyzed single‐cell data from 4 CRC patients who received anti‐PD‐1 antibody treatment and achieved CR/PR (GSE236581) (Figure S2E,F and Table S2).

The results revealed immunotherapy enhanced the expression of not only IgG but also SSR4 in tumor‐infiltrating CD19^+^ B cells (Figure 2F and Figure S2G); meanwhile, SSR4‐high plasma cells (PC) established markedly enhanced interactions with multiple effector CD8^+^, CD4^+^ T‐cell, and stromal cell subsets, compared with SSR4‐low PC (Figure 2G and Figure S2H). These results suggested that SSR4 may be involved in the development of B cells into plasma cells and their immune functions through regulating the transport and glycosylation of some proteins in the ER in CRC.

### 2.3 SSR4^hi^CD19^+^ B Cells in TLS are Positively Correlated With Favorable Prognosis in CRC

**FIGURE 3:**
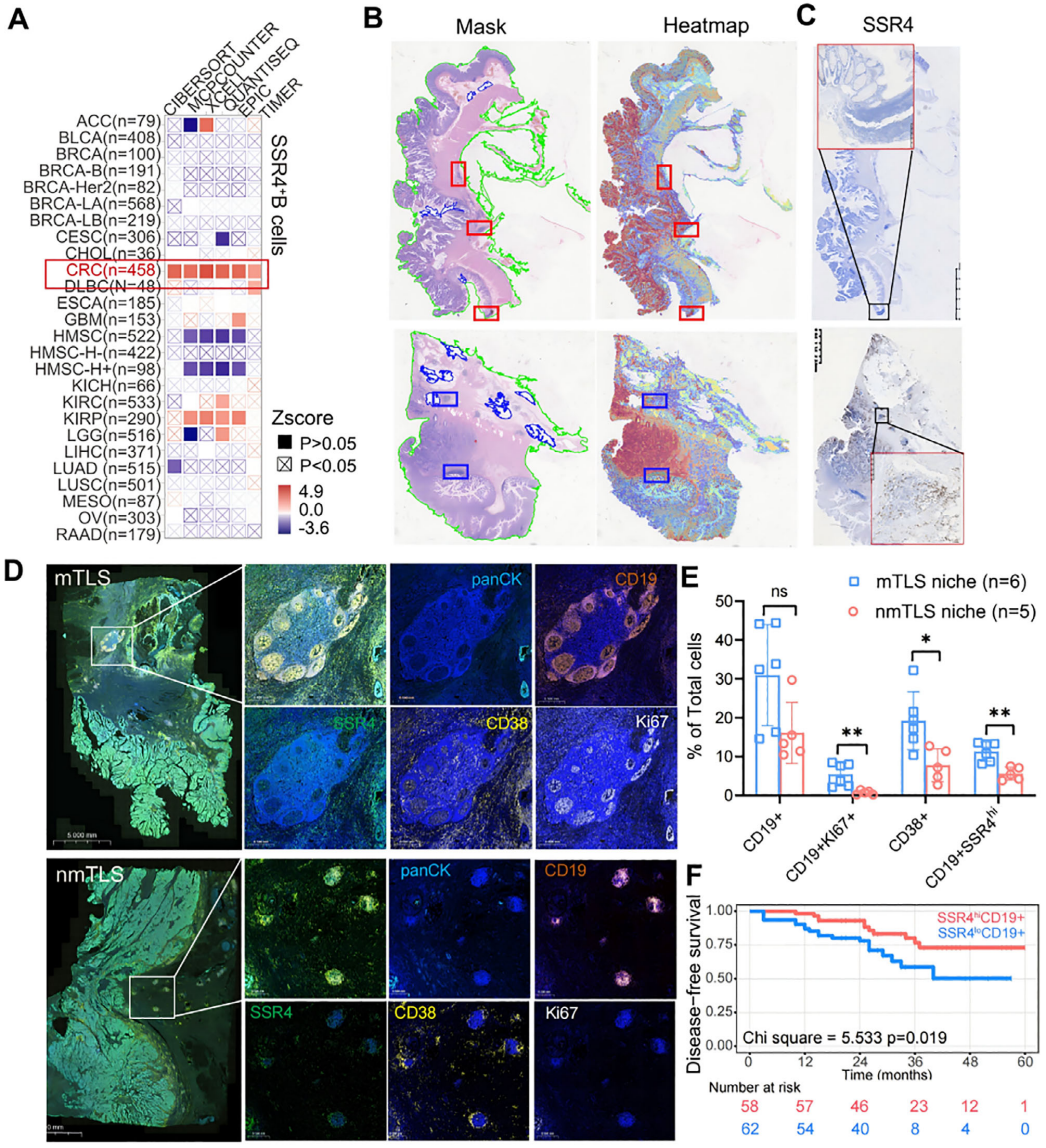
High SSR4 expression in B cells correlates with favorable prognosis and immune‐active tertiary lymphoid structure (TLS) in colorectal cancer (CRC). (A) A variety of immunoinfiltration algorithms were used to analyze the clinical correlation between the infiltration SSR4^+^B cells in different types of TCGA tumors and the prognosis of different tumors. (B) Application of the CLAM (clustering‐constrained attention multiple instance learning) model to 299 whole‐slide images (WSIs) of CRC for recurrence prediction. Model‐derived attention heatmaps delineated regions contributing to recurrence risk, with pathologist‐annotated TLS showing heterogeneous relevance: “red” high‐attention TLS associated with higher recurrence risk, and “blue” low‐attention TLS linked to lower risk. Model performance was assessed using 10‐fold cross‐validation; representative attention heatmap images are shown. (C) Integration of SSR4 immunohistochemical (IHC) staining with model heatmaps showed that “red” high‐risk TLSs exhibited low SSR4 expression, while “blue” low‐risk TLS displayed high SSR4 expression, indicating that the functional state of TLS, potentially represented by SSR4 abundance, influences recurrence outcomes. (D) Representative Multiplex immunofluorescence (mIF) images of panCK, CD19, SSR4, CD38, Ki67 in non‐mature and mature lymphoid structure (TLS) niche from human colorectal cancer tissues (n = 11 tissue samples from 10 CRC patients). Representative mIF images are shown. (E) Quantitative analysis of B cell subtypes in mTLS and nmTLS. Group comparisons were performed using the unpaired two‐tailed Student's *t*‐test. data are presented as mean ± standard deviation (SD) (^*^ *p *< 0.05, ^**^ *p *< 0.01). (F) The Kaplan–Meier (KM) survival curve analysis the correlation between the expression of SSR4 in TLS and prognosis of patients with CRC (n = 120); *p* values were calculated by the log‐rank test.

Analysis results of he Gene Expression Profiling Interactive Analysis (GEPIA) database showed that the SSR4 expression levels of memory B cells and plasma cells in normal tissues were significantly higher than those in CRC tissues (Figure S3A). Using a multivariate Cox proportional hazards model, we further examined the relationship between SSR4 expression in CD19^+^ B cells and prognosis across different cancer types. The results demonstrated a statistically significant positive correlation between high SSR4 expression in B cells and improved prognosis in CRC patients (n = 458) (Figure 3A).

The CLAM (clustering‐constrained attention multiple instance learning) model was applied to 299 whole‐slide images (WSIs) of CRC to predict tumor recurrence. Ten‐fold cross‐validation yielded a mean AUC of 0.685 ± 0.065, indicating stable predictive performance (Figure S3B). Spatial heatmaps generated by the model delineated distinct regional contributions. Manual annotation of tertiary lymphoid structure (TLS) revealed heterogeneous predictive relevance: some TLS appeared as “red” high‐attention zones associated with elevated recurrence risk, whereas others were “blue” low‐attention zones linked to lower risk (Figure 3B). Integration with SSR4 immunohistochemical (IHC) staining showed that TLS regions highlighted as “red” on heatmaps generally exhibited reduced SSR4 expression, whereas “blue” low‐attention TLS displayed higher SSR4 levels (Figure 3C). These findings suggest that the immune functional state of TLS, potentially reflected by SSR4 abundance, plays a crucial role in recurrence prediction.

Additionally, multiplex immunohistochemical (mIHC) staining results showed that the levels of plasma cells (CD38^+^) and CD19^+^SSR4^hi^ B cells were higher in mature TLS niche (CD19^+^Ki67^+^ mTLS, n = 6) compared to immature TLS niche (CD19^+^Ki67^−^ nmTLS, n = 5) (Figures 3D,E and Figure S3C), which suggested that SSR4 expression may be associated with TLS maturity. We further analyzed 120 CRC tissues containing TLS collected from our hospital between January 2018 and December 2019, and Kaplan–Meier survival analysis further showed that patients with SSR4^hi^CD19^+^ in TLS had a significantly longer disease‐free survival (DFS) (Figure 3F and Figure S3D,E). These findings highlight that SSR4^hi^CD19^+^ B cells within TLS are positively correlated with a favorable prognosis in CRC.

### 2.4 SSR4 is Involved in Peripheral B‐cell Mediated Immune Response

**FIGURE 4:**
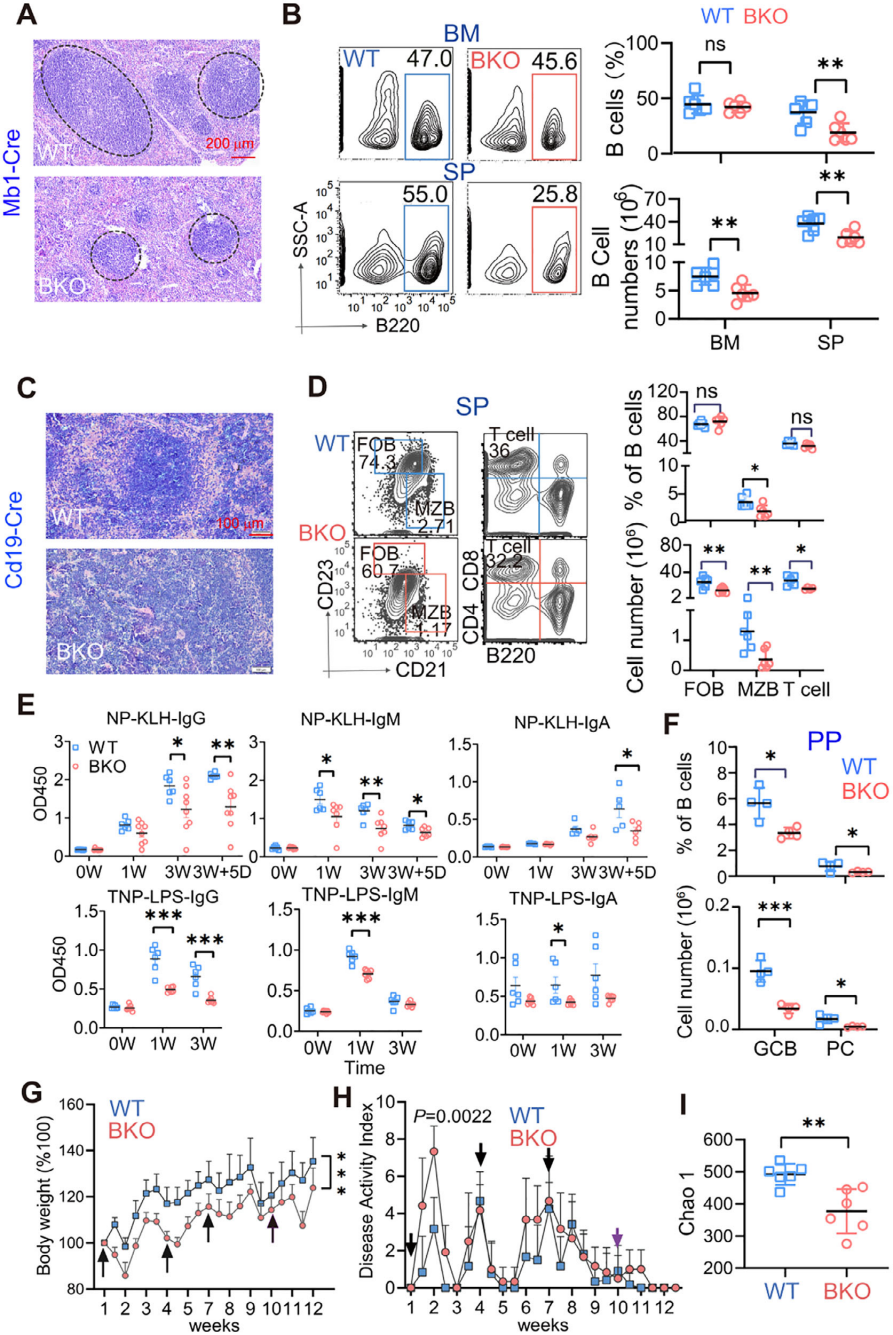
B cell–specific deletion of SSR4 impairs germinal center formation, antibody production, and intestinal TLS‐mediated mucosal protection. (A) H&E staining observes the difference of splenic germinal center between the immunized adult wild‐type (*Mb1*‐Cre) and *Ssr4* ^BKO^ mice (8–10 weeks old).Representative images are shown. (B) Flow cytometry analyses of B220^+^ B cells in the bone marrow and spleen of mice (*Ssr4* ^BKO^; *Mb1*‐Cre, 8–10 weeks old). (C) H&E staining observes the difference of splenic germinal center between the immunized adult *Ssr4* ^BKO^ (*Cd19*‐Cre) and wild‐type mice (8–10 weeks old). Representative images are shown. (D) Flow cytometry analyses of follicular (FO) B cell, marginal zone (MZ) B and T cell in the spleen of wild‐type and *Ssr4* ^BKO^ mice (8‐10 weeks old, n = 6 per group). Representative plots and summary graphs are presented. (E) ELISA of NP/TNP‐specific antibody isotypes in the serum of wild‐type and *Ssr4* ^BKO^ mice (8‐10 weeks old) immunized i.p. with NP‐KLH (n = 6) and TNP‐LPS (n = 6). (F) Flow cytometry analyses of germinal center B cells (GCB) and plasma cells (PC) in the Peyer patch (PP) of wild‐type (n = 4) and *Ssr4* ^BKO^ mice (8‐10 weeks old; n = 4 per group). Representative plots and summary graphs are presented. The body weight (G) and disease activity index (DAI) (H) of wild‐type (n = 12) and *Ssr4* ^BKO^ (n = 12) mice in the DSS‐induced colitis model. (I) Alpha diversity analysis of commensal microbiota in the fecal extracts of age‐matched wild‐type and *Ssr4* ^BKO^ (n = 6 per group) mice using 16S rRNA sequencing. Comparing two groups, data were analyzed using the two‑tailed Student's *t*‑test. Results are presented as mean ± SD. Statistical significance is indicated as ^*^ *p* < 0.05, ^**^ *p* < 0.01, ^***^ *p* < 0.001.

To further explore the functional role of SSR4 in B cell biology and TLS formation, we crossed *Ssr4*‐floxed mice with *Cd19*‐Cre (expressing the Cre recombinase predominantly in peripheral B cells) or *Mb1*‐Cre (strongly expressing the Cre recombinase in early stages of bone marrow B cells) mice (Figure S4A,B). Deletion of SSR4 using the *Mb1*‐Cre did not significantly reduce the number of Pre‐Pro B, pro‐B, pre‐B, and immature B cells (Figure S4C,D) in bone marrow, interestingly, smaller germinal centers and a sharp decrease in B220^+^cells were observed in the spleen of *Ssr4*‐knockout mice (Figure 4A,B). So, we examined the role of SSR4 in regulating peripheral B cells by using the *Ssr4* ^BKO^ mice generated with *Cd19*‐Cre, and we used this BKO model for the following studies. Compared with wild‐type (WT) control mice, the *Ssr4* ^BKO^ mice had a smaller splenic germinal center, a decreased frequency and absolute number of follicular B (FOB), marginal zone B (MZB) cells in spleen (Figures 4C,D), and lower levels of serum specific IgG and IgM (*p *< 0.01) after immunization with NP‐KLH (T‐cell‐dependent antigen) and NP‐LPS (T‐cell‐independent antigen). Although antigen‐specific IgA levels exhibited a modest but statistically significant reduction, the magnitude of decrease was less pronounced compared with IgG and IgM (Figure 4E).

In intestinal chronic inflammation, B cells exert protective effects on the intestinal mucosa by producing immunoglobulins to neutralize and clear intestinalogens. Moreover, the local TLS can limit pathogens or self‐antigens to the area and prevent systemic dissemination. Given roles of B cells in the intestinal mucosal barrier and the reduction in the number of germinal center B (GCB) cells and plasma cells (PC) in the Peyer's patches (PP) in the intestine of *Ssr4* ^BKO^ mice (Figure 4F), we validated the impact of *Ssr4* deletion on B cell‐mediated inflammation using the DSS‐induced colitis model (Figure S4E). Compared with WT control mice with colitis, the *Ssr4* ^BKO^ mice with colitis have lighter weights and a higher disease activity index (DAI) (Figures 4G,H). Additionally, the alpha diversity index of *Ssr4* ^BKO^ mice was significantly lower compared to the wild‐type group (*p* < 0.01) after DSS treatment, which suggested that *Ssr4* deletion in B cells leads to an imbalance in the intestinal microenvironment and a trend toward a single structure of the microbiota (Figure 4I and Figure S4F). These findings indicate that *Ssr4* deletion reduces B‐cell mediated immune response and the formation of intestinal mucosal TLS, resulting in the barrier protective effect of B cells on the intestinal mucosa more susceptible to disruption by exogenous inducers.

### 2.5 SSR4 Mediates the N‐glycosylation Modification of BAFFR in B Cells

**FIGURE 5:**
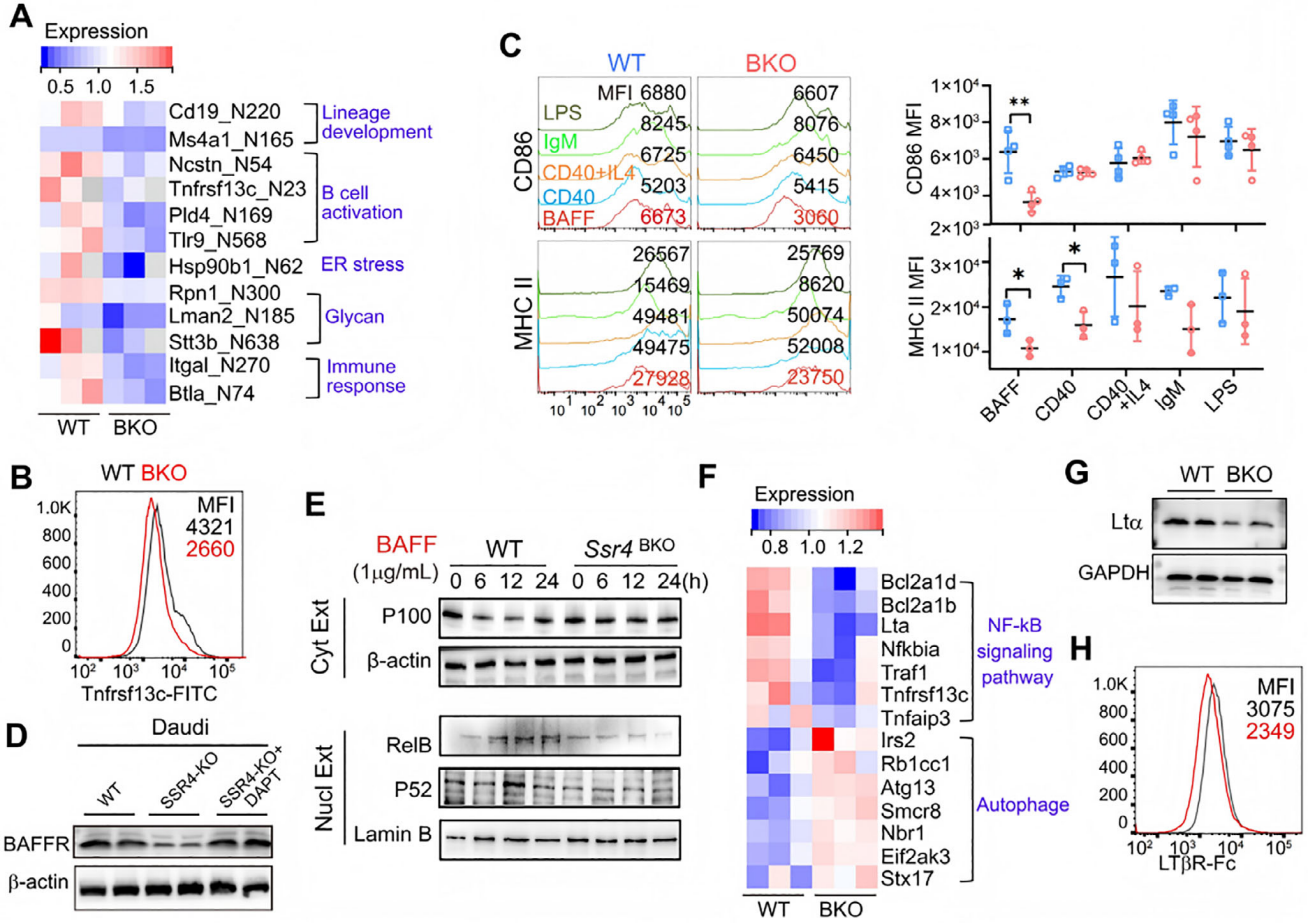
SSR4 deficiency affects B cell activation through NF‐kB signaling pathway. (A) The heatmap showing the down‐regulate N‐linked glycosylation modification site between *Ssr4* ^BKO^ B cells and wild‐type‐B cells (three biological repetitions). (B) Flow cytometric analyses of the indicated markers on gated CD45^+^CD138^+^ B cells from adult *Ssr4* ^BKO^ and wild‐type mice (8–10 weeks old). Data are presented as representative histograms. (C) Flow cytometric analyses of the indicated activation markers CD86 (n = 4) and MHC II (n = 3) on gated *Cd19* ^+^ B cells from unimmunized adult wild‐type and *Ssr4* ^BKO^ mice (8–10 weeks old), presented as representative histograms (left) and summary graph of median fluorescence intensity (MFI) (right). (D) Immunoblot analysis of the indicated proteins using cytoplasmic of wild‐type (WT) and SSR4‐KO Daudi cell line or stimulated with g‐secretase inhibitor (DAPT). Representative blots are shown. (E) Immunoblot analysis of indicated proteins in cytoplasmic or nuclear extracts of BAFF‑stimulated splenic B cells. Representative blots are shown. (F) The heatmap showing the results of BAFF‐stimulated transcriptome sequencing of major differential genes after SSR4 knockout in B cells. (G) Immunoblot analysis of the indicated proteins using cytoplasmic of wild‐type (WT) and *Ssr4* ^BKO^ splenic *Cd19* ^+^ B cells. Representative blots are shown. (H) Flow cytometric analyses of the LTβR on gated *Cd19* ^+^ B cells from unimmunized adult wild‐type and *Ssr4* ^BKO^ mice (8–10 weeks old). Representative histograms are shown. For (A) and (F), differential expression analysis was performed using the two‑sided Wilcoxon rank‑sum test; statistical significance was defined as *p* < 0.05. For (C), group comparisons were performed using the two‑tailed Student's t‑test; data are presented as MFI (mean ± SD); ^*^ *p* < 0.05, ^**^ *p* < 0.01.

As an important part of a translocation channel in the ER of eukaryotic cells, the TRAP complex is not only related to co‐translational protein translocation, but can also regulate biological processes such as N‐glycosylation, calnexin, and unfolded protein response [10]. The SSR4 (TRAP δ) subunit is associated with a congenital disorder of glycosylation (*Ssr4* CDG) wherein the X‐linked SSR4 gene is mutated [17], however, the mechanism by which SSR4 regulates glycosylation during the differentiation of B cells remains unclear. The quantitative analysis of the protein profile revealed that the knockout of SSR4 affected the transport process from the Golgi apparatus to the ER; further analysis showed that the knockout of *Ssr4* had no effect on the protein and mRNA expression levels of other members of the TRAP family (Figure S5A–F). The glycoproteomic analysis results of mouse B220^+^ B cells showed that *Ssr4* knockout altered glycosylation levels, potentially affecting B cell lineage development (*Cd19*_N220 and *Ms4a1*_N165), B cell activation (*Ncstn*_N54, *Tnfrsf13c*_N23, *Pld4*_N169 and *Tlr9*_N568), ER stress (*Hsp90b1*), Glycan (*Rpn1*_N300, *Lman2*_N185 and *Stt3b*_N638), and immune response (*Itgal*_N270 and *Btla*_N74) (Figure 5A; Figure S5G,H and Table S3). Further studies have supported that SSR4 knockout can reduce the protein level of BAFFR (Tnfrsf13c) on the surface of B cells and inhibit B cell activation induced by BAFF (Figures 5B,C).

As a ubiquitous intramembrane cleavage protease, γ‐secretase can shed the ectodomain of some receptors with aberrantly glycosylated, such as BCMA. Consistent with the results of mouse B cells, SSR4 knockout in Burkitt's lymphoma Daudi cells significantly reduced the expression of BAFFR (*Tnfrsf13c*) proteins, which was restored by treatment with the γ‐secretase inhibitor (DAPT) (Figure 5D). Remarkably, the SSR4 deficiency attenuated BAFF‐induced non‐classical NF‐kB activation, as revealed by the impaired loss of cytoplasmic p100 and induction of nuclear p52 and RelB (Figure 5E).

To further elucidate the effect of SSR4 deficiency, transcriptomic analysis was performed on B cells stimulated with BAFF. Heatmap results showed that *Ssr4* knockout inhibited NF‐κB signaling pathway while promoted the expression of autophagy‐related genes, especially, the deletion of SSR4 in mouse B cells suppressed the expression of pro‐activation genes Lta/Traf1, anti‐apoptotic genes Bcl2a1d/b, and anti‐overactivation genes Nfκbia/Tnfaip3 (Figure 5F). Given the role of B cell‐derivedcc lymphotoxin α (LTα) and membrane‐bound heterotrimeric (LTα1β2) complex in inflammation‐related immune diseases, as well as its importance in B cell development and lymphoid organ formation [18], the further study revealed SSR4 knockout suppressed the expression levels of LTα protein and its surface LTα1β2 (detected by LTβR‐Fc) in mouse B cells (Figures 5G,H).

The above results indicate that SSR4 deficiency in B cells reduces the glycosylation level of N23 on BAFFR, inhibits BAFF/BAFFR‐NF‐κB signaling in B cells, and decreases the protein level of LTα1β2 on the B cell surface, which may ultimately impair the TLS formation in the microenvironment.

### 2.6 SSR4 maintains Homeostasis of ER Stress and Autophagy in Plasma Cells Through Binding DDOST

**FIGURE 6:**
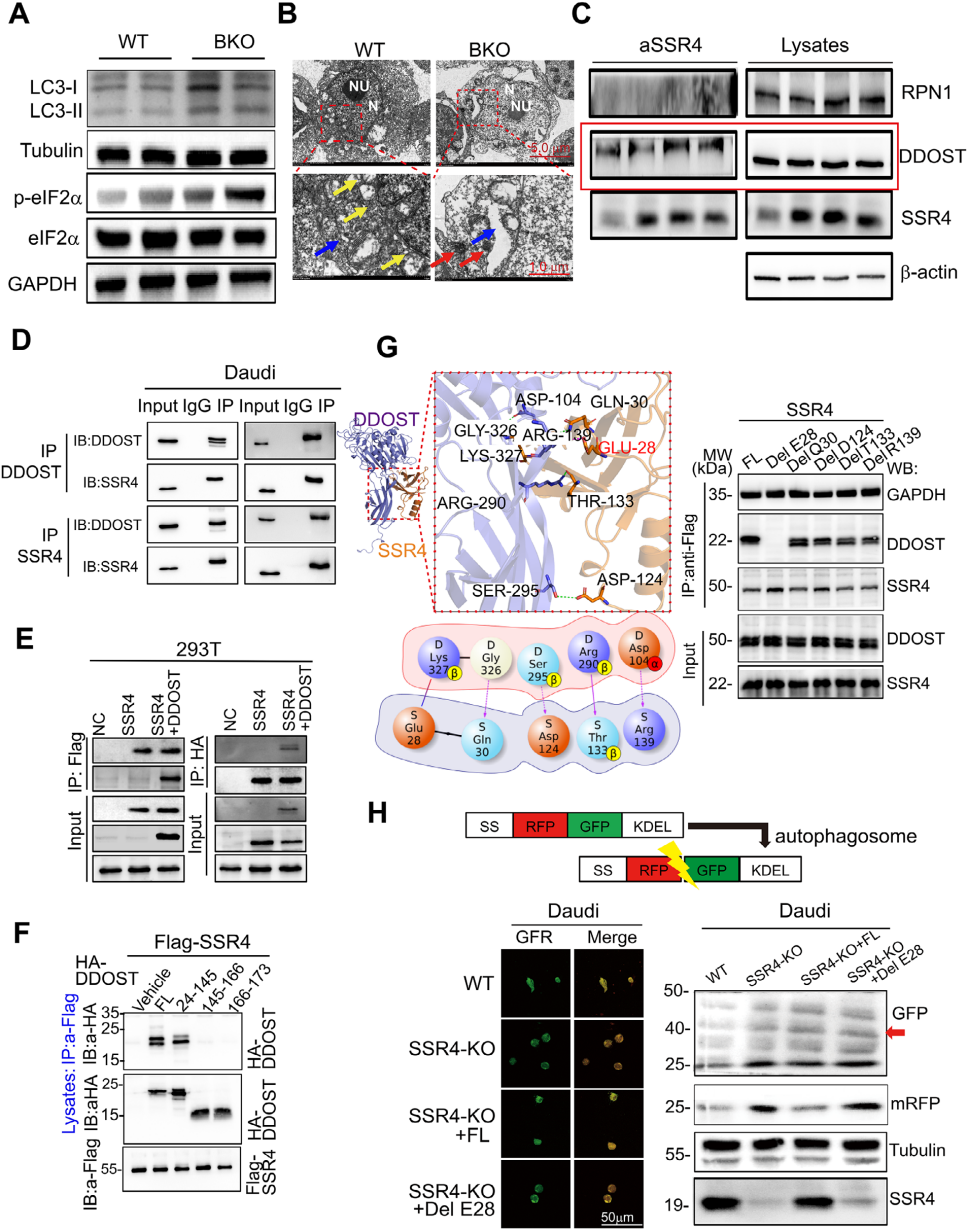
SSR4 maintains homeostasis of ER stress and autophagy in plasma cells through binding DDOST. (A) Western blot analysis of p‐eIF2α, LC3 in primary mouse plasma cells. Representative blots are shown. (B) Transmission electron microscopy (TEM) revealed ER lumen expansion, membrane rupture, and increased autophagosome formation in SSR4‐deficient cells. Co‐immunoprecipitation (Co‐IP) assays in mouse B cells (C), Daudi (D), and 293T (E) cells confirmed a direct physical interaction between SSR4 and DDOST. (F, G) Domain‐mapping and mutagenesis studies based on molecular docking analysis (docking score = ‐266.39 kcal/mol; confidence = 0.9217) identified glutamate 28 (E28) of SSR4 as the key residue mediating DDOST binding. Co‐IP experiments validated that mutation of E28 (E28A) abolished the SSR4‐DDOST interaction. (H) Confocal microscopy of autophagic activity using the mRFP‐GFP‐LC3 reporter system showed elevated autophagosome formation (increased mRFP/mRFP‐GFP ratio) in SSR4‐deficient cells. Reintroduction of full‐length SSR4 rescued this phenotype, whereas the E28A mutant failed to do so, confirming the functional importance of the SSR4‐DDOST interaction in maintaining ER homeostasis, n = 3.

While B cells become plasmablasts and are fully efficient in Ig secretion, the cells undergo massive changes in their organelles, including Ig processing is overwhelmed into the ER lumen, resulting in an increased level of unfolded and misfolded proteins with a high ER stress state [19]. And, to prevent excessive cellular damage caused by ER stress, which indirectly regulates plasma cells autophagy, TRAP complex regulates quality control of N‐glycosylation during ER stress [14], but how SSR4 regulates the ER stress‐autophagy axis of plasma cells is unknown. As shown in Figure 6A, SSR4 knockout increased the levels of ER stress (p‐eIF2α) and autophagy (LC3‐II) in mouse plasma cells, and electron microscopy revealed ER structural expansion and membrane damage in SSR4 knockout cells, along with increased autophagosome formation (Figure 6B) in mouse plasma cells.

The SSR4 may interact with some OST subunits, and its deficiency leads to OST dysfunctionality. The STRING database predicts an interaction between SSR4 and the DDOST subunit of OST, which is essential for complex stability (Figure S6A,B). We then performed coimmunoprecipitation (Co‐IP) with an anti‐SSR4 antibody in mouse B cells, Daudi, and 293T cells, and further supported that SSR4 can bind to the DDOST subunit of the OST complex (Figures 6C‐E).

To briefly describe the structural domains in which SSR4 and DDOST interact, the plasmids of specific Flag‐SSR4‐truncated or point mutation were constructed to transfect 293T cells based on molecular docking results (a docking score of −266.39 kcal/mol and a confidence score of 0.9217) (Figure S6C and Table S4). The results of Co‐IP experiments indicated that the glutamate at position 28 (E28) of SSR4 plays a crucial role in its binding with DDOST (Figures 6F,G). In addition, confocal microscopy revealed distinct red fluorescent signals indicative of autophagic activity in SSR4‐knockout cells. This autophagic response was effectively rescued by the introduction of a full‐length SSR4 plasmid, but remained unaffected upon transfection with the E28 point‐mutant plasmid. Consistent with these observations, Western blot analysis further demonstrated an increase in ER‐associated autophagy following SSR4 knockout, as demonstrated by a shift in the mRFP/mRFP‐GFP fluorescence ratio (Figure 6H and Figure S6D).

These findings demonstrated that SRR4 assists in the glycosylation modification activity of OST complexes by binding DDOST, thereby maintaining the homeostasis of ER stress autophagy axis in plasma cells.

### 2.7 *Ssr4* ^BKO^ Promotes Spontaneous Intestinal Adenoma in *Apc^m^* ^in/+^ Mice

**FIGURE 7:**
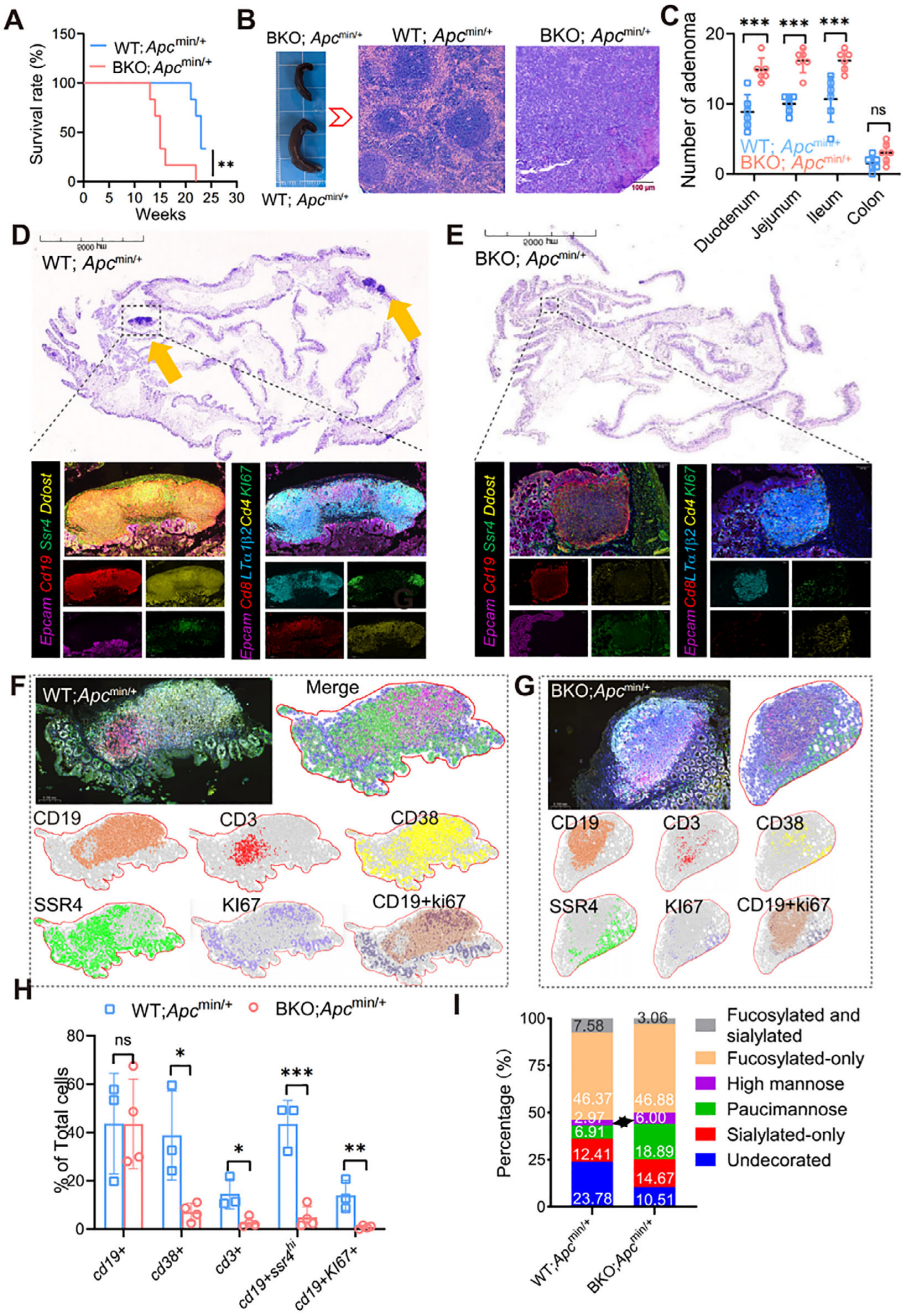
SSR4 deficiency in B cells promotes adenoma progression and impairs TLS formation in *Apc* ^Min/+^ mice. (A) Kaplan–Meier survival analysis of WT;*Apc* ^min/+^ mice (n = 6) compared with BKO;*Apc* ^min/+^ (n = 6) controls. Survival analysis was performed using the Kaplan–Meier method, with group differences assessed by the two‐sided log‐rank test (^**^ *p* < 0.01). (B) Representative images of spleen from two groups (left) and its H&E staining images (right). (C) Number of intestinal adenomas in different groups WT;*Apc* ^min/+^ (n = 6) and BKO;*Apc* ^min/+^ (n = 6) mice, each circle represents a mouse. (D, E) Histological morphology of colorectal tissues in two groups of animals, yellow arrows indicate the formation of mature tertiary lymphatic structures; down is multi‐immunofluorescence of *Epam*, *Cd19*, *Ssr4*, *Ddost*, *Cd8*, *LTα1β2*, *Cd4* and *ki67* in the TLS of colon tissue in two groups are shown. (F, G) Representative multiplex immunofluorescence (mIF) staining was performed to identify TLS in WT;*Apc* ^min/+^ and BKO;*Apc* ^min/+^ mice model. (H) Quantitative statistical analysis of TLS between the WT;*Apc* ^min/+^ (n = 3) and BKO;*Apc* ^min/+^ (n = 4) mice model. (I) LC‐MS/MS analysis was performed once with pooled serum from 3 mice per group to ensure adequate Ig quantity for analysis. Data are presented as LC‐MS/MS profiles. For (B) and (H), Group comparisons were performed using the two‐tailed Student's *t*‐test; data are presented as mean ± SD (^*^ *p *< 0.05, ^**^ *p *< 0.01, ^***^ *p *< 0.001).

To elucidate the effect of SSR4 on the immune function of B cells in CRC susceptibility and tumor progression, we constructed *Ssr4* ^BKO^;*Apc* ^min/+^ mice (Figure S7A,B). We found that BKO;*Apc* ^min/+^ mice led to decreased survival rate in mice and disrupted germinal centers in the spleen (Figure 7A,B). The results revealed that the number of adenomas in the BKO;*Apc* ^min/+^ mice increased significantly compared to WT;*Apc* ^min/+^ mice (Figure 7C). Moreover, pathological observation revealed that the intestinal tissues of BKO;*Apc* ^min/+^ mice exhibited typical fusion of the intestinal wall to form a co‐mural structure, a significant increase in pathological nuclear pleomorphism of epithelial cells, and hyperplasia of the intestinal epithelial layer, and that the colorectal tissue in BKO;*Apc* ^min/+^ group displayed moderately to poorly differentiated adenocarcinoma, while the adenomas in the WT;*Apc* ^min/+^ group were primarily characterized by atypical hyperplasia (Figure S7C).

Multicolor immunofluorescence (mIHC) staining revealed that the TLS in the intestinal tissue of BKO;*Apc* ^min/+^ mice were smaller, lacked distinct light and dark zones, and primarily contained *Cd19* ^+^ and *LTα1β2* ^+^ B cells. The expression levels of SSR4 were reduced, and the number of other immune cells, particularly *Cd4* ^+^ T cells, was also lower in the SSR4 knockout group (Figures 7D,E and Figure S7D). In the mouse model, we further verified the effect of SSR4 deficiency on TLS maturation. Compared with WT;*Apc* ^Min/+^mice, the proportions of *Cd38* ^+^ plasma cells, *Cd19* ^+^ *Ssr4* ^hi^ cells, and *Cd19*+*Ki67* ^+^ cells in TLS of BKO;*Apc* ^Min/+^ mice were extremely significantly reduced (*p *< 0.05). The proportions of *Cd3* ^+^ cells were also significantly decreased (*p *< 0.05), confirming that SSR4 deficiency directly leads to impaired recruitment and activation of core immune cells in TLS (Figures 7F‐H). The formation of TLS in colorectal tissues was further compared between the two different types of mice models. In the BKO;*Apc* ^min/+^ group, seven lymphocyte aggregates were detected in four of six mice. In the WT;*Apc* ^min/+^ group, six lymphocyte aggregates were observed in three of six mice. No significant difference in the total area of TLS was observed between the two groups (Figure S7E).

The LC‐MS/MS analysis results of serum immunoglobulins (Ig) showed that high mannose accounted for a relatively higher proportion in the BKO;*Apc* ^min/+^ group than those in the WT;*Apc* ^min/+^ group (Figure 7I and Figure S8A,B). Glycoprofiling of serum Ig revealed that Ig predominantly exhibited the HexNAc(3)Hex(1)Fuc(1) glycosylation pattern in the BKO;*Apc* ^min/+^ group, whereas no such glycopeptides were detected in the WT;*Apc* ^min/+^ group (Figure S8C).

The above results indicate that SSR4 can not only regulate the effect of plasma cell antibody preparation through ER stress autophagy axis and Ig glycosylation modification, but also promote the TLS formation in TME by regulation the LTα1β2 levels on the B cell surface.

### 2.8 Overexpression of SSR4 Enhances the Anti‐tumor Effect of Antibodies Produced by CHOS Cells

**FIGURE 8:**
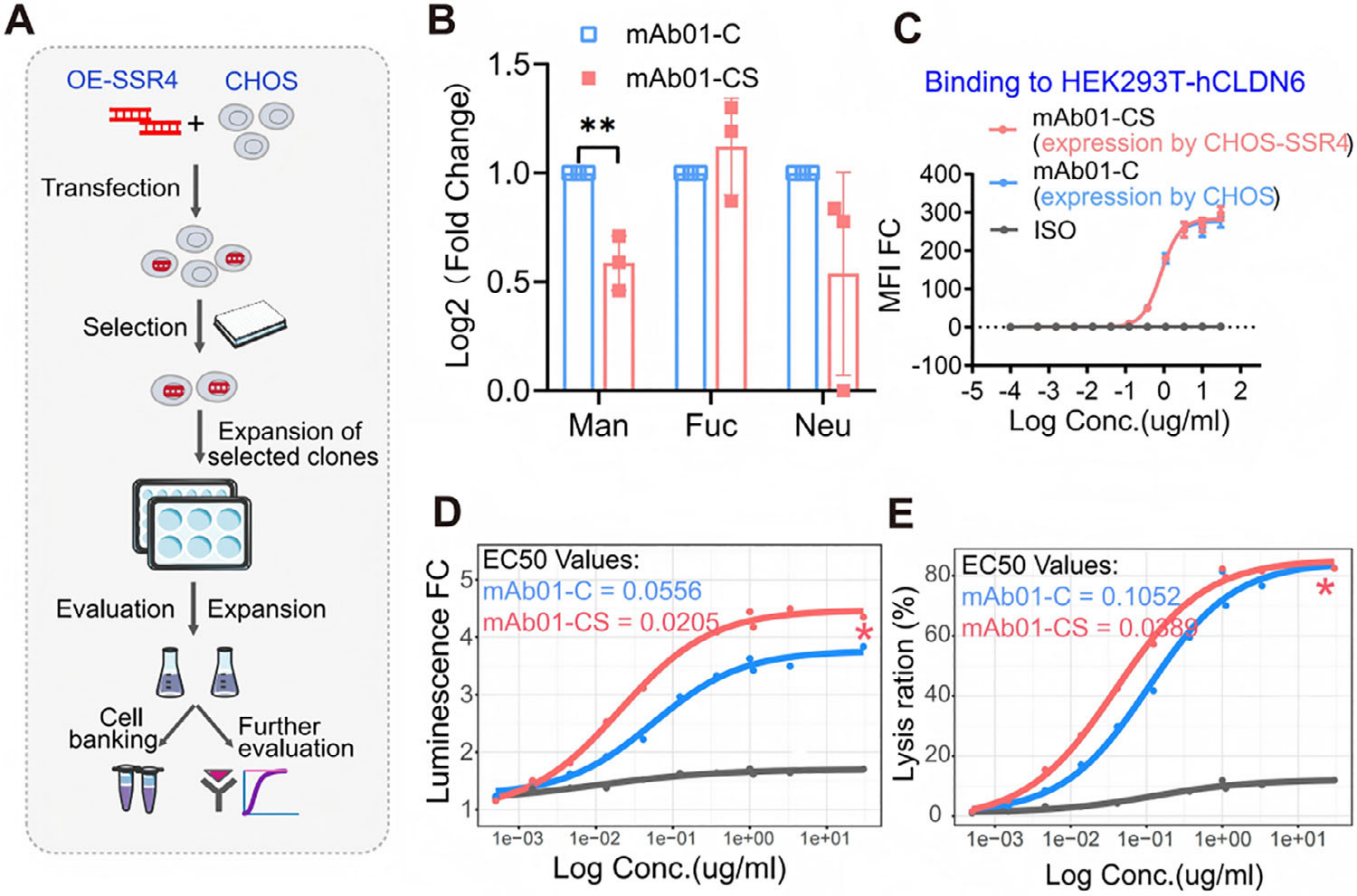
Overexpression of SSR4 enhances the anti‐tumor effect of antibodies produced by CHOS cells. (A) Flow chart of monoclonal antibody obtained by SSR4 overexpression of CHOS cell line. (B) The histogram shows the effect of SSR4 overexpression on N‐glycosylation modification of monoclonal antibody at the glycosylation site (Asn297) of monoclonal antibody (mAb01‐CS vs. mAb01‐C). Data are presented as log2 (fold change) relative to mAb01‐C, with mean ± SD from n = 3 independent biological replicates. Statistical significance was assessed using a two‐tailed Student's *t*‐test. ^**^ *p* < 0.01. (C) Antibody binding assay of CHOS expression was detected by flow cytometry. ISO is a human IgG1 control antibody. Data are presented as fold change in mean fluorescence intensity (MFI) relative to control across increasing antibody concentrations (log scale). (D) The antibody‐dependent cell‐mediated cytotoxicity (ADCC) experiments were performed by HEK293T‐hCLDN6, ISO is a human IgG1 control antibody. Data are presented as fold change in luminescence relative to control. (E) The complement dependent cytotoxicity (CDC) experiments were performed by HEK293T‐hCLDN6, specific lysis was calculated. For both ADCC and CDC functional assays, dose‐response curves were fitted by nonlinear regression analysis based on a four‐parameter logistic (4PL) model. The half‐maximal effective concentration (EC50) was calculated to evaluate the functional potency of different antibody groups. Extra sum‐of‐squares F‐test was used to compare overall differences in dose‐response curves and EC50 values between mAb01‐C and mAb01‐CS groups.

To further evaluate the impact of SSR4 on the quality of antibody, SSR4 was overexpressed in Chinese hamster ovarycells (CHOS, endogenous SSR4‐deficient) (Figure S9A,B) for anti‐human CLDN6 IgG1 industrial production (Figure 8A). The results of high‐resolution LC‐MS/MS analysis showed that SSR4 overexpression significantly reduced high‐mannose (HM, Man5‐9) glycoforms at Asn297 of IgG1 (mAb01‐CS) to 0.59 ± 0.12 fold that of the control group (mAb01‐C) (*p* < 0.05), while maintaining comparable levels of core fucosylation (Fuc) and sialylation (Neu) (Figure 8B).

To exclude the potential influence of antigen binding capacity, we further determined the binding affinity of ISO, mAb01‑C, and mAb01‑CS against CLDN6. No significant difference in antigen binding was observed among all groups (Figure 8C), indicating that SSR4‑mediated glycosylation modification did not interfere with antibody‐antigen recognition (Figure 8C and Figure S9C).

The half‐maximal effective concentration (EC50) was calculated to evaluate antibody potency. In the ADCC assay, the EC50 values of mAb01‐C (expression by CHOS) and mAb01‐CS (expression by CHOS‐SSR4) were 0.0556 and 0.0205, respectively. In the CDC assay, the EC50 values were 0.1052 for mAb01‐C and 0.0389 for mAb01‐CS. Collectively, mAb01‐CS exhibited markedly lower EC50 and superior functional potency than mAb01‐C in both ADCC and CDC modalities. Notably, the magnitude of potency improvement mediated by structural modification was comparable between ADCC (∼2.71‐fold) and CDC (∼2.70‐fold) responses. (Figure 8D,E).

These findings define SSR4‐mediated glycosylation remodeling as a mechanism that reduces immunosuppressive high‐mannose glycans without compromising structural integrity, thereby enhancing FcγR‐mediated effector functions and synergizing with adaptive antitumor immunity, providing a mechanistic basis for SSR4 as a critical regulator of therapeutic antibody optimization.

## 3 Discussion

This study identifies SSR4^high^ B cells as a key B cell subset enriched in colorectal cancer (CRC) tissues, correlating with improved immunotherapy responses and favorable prognosis. Using B cell‐conditional *Ssr4*‐knockout (KO) mice (*Ssr4* ^BKO^) mice, we reveal an unexpected role of SSR4 in B cell lineage development and immunoglobulin production, with its deletion impairing proper glycosylation of BAFFR via the NF‐kB signaling pathway. Mechanistically, SSR4 interacts with N‐glycosyltransferase DDOST, regulating ER autophagy through post‐translational modifications (PTMs). These findings demonstrate that SSR4 acts as a pivotal regulator of immune microenvironment (IME) remodeling through a two‐pronged role: it facilitates mature TLS (mTLS) assembly by controlling B cell‐surface LTα1β2, and fine‐tunes the output and glycan composition of antibodies by plasma cells (SCHEME).

Consistent with previous studies [11, 20], we identify SSR4^high^ plasma B cells as positively associated with immunotherapy response in CRC. Spatial analyses further reveal their enrichment within mature TLS, which has been implicated in antigen presentation and humoral immune optimization [21, 22, 23]. However, the precise mechanism by which SSR4 governs B cell development, differentiation, and TLS formation remains unclear.

Humoral immunity holds significant potential for B‐cell‐targeting immunotherapy, with B lymphocytes serving as pivotal mediators of this immune response [24, 25, 26]. Delving into the lineage developmental characteristics of B lymphocytes is essential for a deeper understanding of their roles in tumor immunity [15, 27, 28]. In our study, we demonstrated that SSR4 is essential for maintaining normal B‐cell lineage development and activation. Additionally, SSR4 deficiency in *Cd19* ^+^ B cells causes aberrant immunoglobulin production and impaired formation of mTLS. These results further support the collaborative role of B cells in mediating both humoral and cellular immunity.

BAFF, also known as CD257, TNFSF13B, or BLyS, is an important B‐cell activating factor that belongs to the tumor necrosis factor (TNF) family [29, 30]. It primarily functions by interacting with three receptors expressed on B cells, BAFFR [31], TACI [32], and BCMA. [33] Our study revealed that SSR4 deletion significantly inhibited BAFF‐stimulated B cell activation and suppressed the glycosylation sites and expression levels of the B cell surface receptors BAFFR (*Tnfrs13c*) and BCMA (*Tnfrsf17*). Furthermore, SSR4 influences non‐canonical NF‐κB signaling pathways, which are the primary pathways involved in B cell activation [18].

Previous studies have suggested that SSR4 is localized in the ER and regulates N‐linked glycosylation modification [34]. Our data further revealed that SSR4 deletion alters glycosylation levels, potentially impacting B cell lineage development, activation, ER stress, Glycan and Immune response. N‐Linked glycans, synthesized by glycosyltransferases, represent one of the most conserved and abundant forms of protein modification within the ER of eukaryotic cells [35, 36]. While growing evidence indicates that lymphocytes development is regulated by glycans [37, 38, 39, 40, 41], the specific role of N‐linked glycosylation in the growth and development of B cells, particularly from the perspective of PTMs, remains largely unexplored.

The TRAP complex regulates N‐linked glycosylation and plays a critical role in ER stress [14]. As an important component of the TRAP complex, SSR4 is expressed at much higher levels in plasma cells cells compared to other members, such as SSR1‐3. Therefore, the unique role of SSR4 and its molecular mechanisms in B cells and PB cells requires further investigation. In this study, we demonstrated that SSR4 regulates B cell autophagy by binding to the N‐linked glycosyltransferase DDOST at the E28 site. DDOST, an essential subunit of oligosaccharyltransferase (OST), catalyzes the transfer of high mannose‐oligosaccharides from lipid‐linked oligosaccharide donors to asparagine residues within the common motif (Asn‐X‐Ser/Thr) of newly synthesized polypeptide chains [42, 43]. Notably, previous studies have reported that congenital deficiencies of SSR4 and DDOST can lead to congenital glycosylation disorders (CGDs) and subsequent humoral immune deficiencies in patients [12, 44, 45]. Therefore, our study provides a new perspective on the role of SSR4 in N‐linked glycosylation modification.

Accumulating evidence highlights the diverse roles of humoral immunity in tumor progression, with B cells serving as crucial regulators of anti‐tumor responses [41, 46, 47]. In this study, we demonstrated that *Ssr4* deficiency in *Apc* ^Min/+^ mice led to reduced survival, increased tumor burden, disrupted germinal centers, and impaired CD8^+^ T cell infiltration into tumor‐associated TLS, underscoring its importance in maintaining immune homeostasis. Additionally, SSR4 loss disrupted humoral immunity, reducing IGHG1 and IGHG3 levels and altering immunoglobulin glycosylation, with increased high‐mannose (HM) modifications and decreased sialylation. These changes may impair antibody stability and Fc‐mediated immune responses, weakening anti‐tumor immunity. Of note, we established the orthotopic colorectal cancer models by cecal injection of MC38‐luc‐puro cells, and our results showed that B‐cell specific depletion of SSR4 promoted tumor progression. However, TLS was not observed in tumor tissues of either WT or *Ssr4* ^BKO^ mice, which may result from differences in the tumor microenvironment between transplanted tumors and spontaneous tumors (data not shown).

Given the pivotal role of glycosylation in antibody structure and function [35], we further analyzed the glycosylation modification profiles at the Asn297 site. Due to the low endogenous expression of SSR4 in CHOS cells, its overexpression effectively reduced immunosuppressive HM glycoforms at Asn297 without altering core fucosylation or sialylation. This selective glycosylation remodeling preserved antibody stability while enhancing FcgR‐mediated effector functions, including antibody‐dependent cellular cytotoxicity (ADCC) and complement‐dependent cytotoxicity (CDC). It means that optimized FcgR interactions not only may improve direct effector functions but may also foster adaptive immune responses within the tumor microenvironment.

This study has several limitations. The formation of TLS is a dynamic process where cellular composition and maturation status can change over time. Assessments based on two‐dimensional histological sections may be influenced by the plane of sectioning, potentially confounding the evaluation of TLS maturity and immune composition. To mitigate the impact of these constraints on the interpretation of our results, this study did not directly investigate the correlation between SSR4 and TLS maturity.

## 4 Conclusion

In summary, our study identifies SSR4 as a key quality control factor in N‐glycosylation during B cell differentiation into plasma cells. In this process, SSR4 regulates the glycosylation modifications of specific molecules (such as BAFFR) and participates in signaling pathways including NF‐κB and endoplasmic reticulum stress‐induced autophagy, thereby promoting the formation of TLS in CRC (SCHEME). Our findings demonstrate that the abundance of SSR4^high^ TLS in the tumor microenvironment is positively correlated with patient survival in colorectal cancer, and antibodies produced in CHOS cells with high SSR4 expression exhibit enhanced anti‐tumor activity. All these findings suggest that SSR4 holds potential translational significance for both clinical diagnosis strategies of colorectal cancer and the production process of immunotherapy antibodies.

## 5 Experimental Section

### 5.1 Mouse Model Construction and Patients’ Specimens

*Ssr4* ^BKO^ mice (produced by crossing *Ssr4*‐flox mice with *Cd19*‐Cre), *Ssr4* ^BKO^;*Apc* ^Min/+^ mice (obtained by crossing *Ssr4* ^BKO^ mice with *Apc* ^min/+^ mice), and the control group mice used in the study were purchased from Nanjing Jicui Yaokang Biotechnology Co., Ltd., China. All mice were raised in the SPF Experimental Animal Center of Nanjing First Hospital and approved by the Animal Ethics Committee of Nanjing First Hospital (Animal Ethics Approval No. SYDW‐2021032). Genomic DNA was extracted from mouse toe tissue for genotyping PCR using the primers listed in Table S5.

All CRC patients received surgical treatment at the Nanjing First Hospital from 2017–2022. All patients were informed of the study and provided informed consent prior to specimen collection Tables S6 and S7. The study was approved by the Ethics Committee of the Nanjing First Hospital (KY20240822‐KS‐06). No clinical study registration number is applicable to this study.

### 5.2 Sequences of RNA Interference (RNAi), CRISPR/Cas9 sgRNA and Primers for qPCR

See Tables S8 and S9.

### 5.3 Spatial Transcriptomic (ST) Data Analysis

ST data from four colorectal cancer tissues containing tertiary lymphoid structures (TLS) were obtained from the HEST database (https://github.com/mahmoodlab/hest) and processed in Seurat (v4.3). Low‐quality spots (library size < 100 UMIs) were removed, followed by normalization, PCA, clustering, and UMAP embedding. TLS regions were annotated by combining H&E image review with TLS‐specific gene signatures, and datasets were integrated using Seurat integration methods to correct for sample‐specific effects. CD19^+^ B‐cell spots were extracted, reclustered with a resolution of 0.2. Differential expression analysis between TLS‐ and non‐TLS‐associated CD19^+^ B cells was performed using the FindAllMarkers function with thresholds of log_2_ fold change >0.25, minimum expression fraction > 0.25, and Benjamini–Hochberg adjusted *p* < 0.05. Significant genes were subjected to pathway enrichment using clusterProfiler (v4.0) with KEGG annotations. Enrichment significance was defined by Benjamini–Hochberg adjusted *p* < 0.05.

### 5.4 Single‐cell RNA Sequencing (scRNA‐seq) Data Analysis

The study retrieved expression matrices from the DISCO (Deeply Integrated human Single‐Cell Omics data) and GEO (Gene Expression Omnibus) databases. Single‐cell RNA sequencing data were analyzed via the Read10X function in the Seurat package. To ensure high data quality, cells with >25% mitochondrial gene expression, < 200 gene counts, or >8,000 gene counts were filtered out. Next, data normalization was done using NormalizeData, and highly variable genes were identified with FindVariableFeatures. Linear dimensionality reduction was performed with ScaleData, followed by principal component analysis (PCA) via RunPCA to select principal components with significant explanatory power for further analysis. For differential expression analysis, FindAllMarkers was used, with significant markers selected by thresholds (e.g., adj. *p* < 0.05). Marker gene expression patterns were visualized using DotPlot and VlnPlot. R v4.2.0 and Seurat v4.4.0 were used throughout, with all analysis parameters documented to ensure result reproducibility. Pseudo‐time analysis was conducted using the Monocle3 package (v1.2.9): the gene expression matrix was extracted via Seurat's GetAssayData, dimensionality reduction via preprocess_cds, batch effect correction via align cds, and further reduction/visualization via UMAP. To clarify the temporal evolution of cell states, the learn graph function was used to infer cell trajectories, and plot‐cell‐trajectory to visualize cell state transition paths on the pseudo‐timeline.

### 5.5 Multiplex Immunofluorescence Staining

Paraffin‐embedded tissue sections were deparaffinized, rehydrated, and subjected to antigen retrieval. After blocking endogenous peroxidase activity and non‐specific binding sites, sections were incubated with primary antibodies (including but not limited to CD19, SSR4, CD38, and Ki67), followed by corresponding fluorescent secondary antibodies and DAPI counterstaining.

Images were acquired by scanning slides using the Leica Aperio VERSA digital pathological image scanner, and three non‐overlapping high‐power fields were randomly selected for each section.

### 5.6 Mouse Immunization and ELISA for Antibody Subtypes

Age‐matched wild‐type and Ssr4BKO mcie (6–8 weeks old) mice were injected intraperitoneally (i.p.) with 0.2 mL of NP‐KLH or TNP‐LPS, blood was collected from the inner canthus at the specified time point, and serum was collected, and subjected to ELISA measurement of NP specific IgM, IgA, IgG. ELISA was used to detect NP/TNP‐specific IgM, IgA, and IgG. The ELISA plate was coated with 500 ng NP‐BSA per well overnight at 4°C. Wash with PBS containing 0.05% Tween 20 and blocking with 1×ELISA Diluent for 1 h, diluted serum was added and incubated for 2 h at room temperature before washing with PBS containing 0.05% Tween 20. HRP‐coupled IgM, IgA, and IgG were added and incubated for 1 h, then washed in turn, and TMB solution was added. After termination of the reaction, absorbance (OD value) was measured at 450 nm wavelength by the enzyme‐labeler.

### 5.7 DSS‐Induced Chronic Enteritis Model

For generating chronic enteritis models, healthy male mice (6–8 weeks old) were subsequently induced by 4 cycles of 2.5% dextran sulfate sodium (DSS) (MP Biomedicals, Santa Ana, CA) in sterile drinking water for 1 week, followed by normal drinking water for another 2 weeks. Body weights were monitored once every 4 days over the duration of the experiment. Symptom severity was assessed once every 4 days beginning at the first cycle of DSS exposure, by symptom score as previously described which included body weight loss (WL) (0  =  < 5%, 1 =  6–10%, 2 = 11–15%, 3 = > 15%); stool consistency (0 = pellet, 2 = pasty, and 4 = diarrhea); occult/gross rectal bleeding assessed by the urine fecal occult blood test kit (0 = negative, 2 = positive, and 4 = gross bleeding). Mice were sacrificed at week 12. Colons tissues were collected, cleaned with PBS, and stored under the required conditions for subsequent analyses.

### 5.8 B‐Cell Purification and In Vitro Analysis

B cells were purified from the splenocytes by anti‐B220‐conjugated magnetic beads (Miltenyl Biotec). Purified B cells in replicate wells of 96‐well plates (2 × 10^5^ cells per well) were stimulated at 37°C with BAFF, anti‐CD40, anti‐CD40+IL‐4, anti‐IgM, and LPS, respectively. After stimulation for 40 h, B cells were collected, and the expression of CD86 and MHC class II was detected by flow cytometry to evaluate B cell activation.

### 5.9 Glycosylated Modified 4DLabelfree Quantitative Proteomics

Splenic B cells (B220^+^CD43^−^) were isolated from *Ssr4* ^fl/fl^;*Cd19*‐Cre^ki/wt^ and *Ssr4* ^fl/fl^;*Cd19*‐Cre^wt/wt^ wild‐type mice. Samples were quickly frozen with liquid nitrogen, stored at −80°C, then thawed on ice, centrifuged at 12,000 g at 4°C for 10 min to remove cell debris. Protein extraction and trypsin digestion. The samples were then transferred to Jingjie Biological for enzyme digestion, enrichment of modified peptides, and Liquid chromatography‐mass spectrometry (LC‐MS) analysis.

### 5.10 Cell Culture and Preparation of Cell Models

The human Burkitt's lymphoma cells Daudi and Raji were purchased from YaJi Biological (China), and cultured in 1640 cell culture medium supplemented with 10% FBS and 1% Penicillin/streptomycin. The CHOS cells for preparing anti‐hCLDN6 IgG1 and the HET‐293 cells with high hCLDN6 expression were obtained from Shanghai GeneChem Co., Ltd. The SSR4‐knockout Daudi monoclonal cell line was constructed using the CRISPR‐Cas9 (Sequences of RNA interference (RNAi), CRISPR/Cas9 sgRNA and primers for qPCR See Tables S8 and S9), and was then transfected with plasmids encoding various SSR4 mutants (DEL145‐165AA, DEL24‐144AA, DEL166‐173AA, Del E28/Q30/D124/T133/R139), respectively, to verify the relevant molecular mechanisms of SSR4. Transmission electron microscopy was used to detect autophagosomes in different cell models.

### 5.11 Detection of Serum Antibody Glycosylation Based on LC‐MS/MS

Serum from mice (BKO;*Apc* ^min/+^ and WT;*Apc* ^min/+^) was collected. 10ul serum was added into balanced beads, incubated at 4°C overnight, and the supernatant was discarded. Washed with 0.5 mL of detergent, removed non‐specific adsorbed protein, and discarded supernatant in triplicate. Followed by SDS‐PAGE and immunoblotting. DTT solution was added to make the final concentration 10 mmol/L, and then reduced in a water bath at 56°C for 1 h. The IAM solution was added to make the final concentration 55 µmol/L, and the reaction was carried out for 40 min away from light. After 4 h of enzyme digestion according to the mass ratio of trypsin to substrate of 1:100, 37°C, the pancreatic enzyme was added, and the enzyme digestion reaction at 37°C was carried out overnight. The peptide was dissolved in 0.1% formic acid by vacuum centrifugation at 45°C and waited for mass spectrometry analysis. The original mass spectrometry files were retrieved separately from the target protein database using Byonic software.

The glycosylation sites of anti‐hCLDN6 IgG1 were identified by LC‐MS. In brief, the 10 mg sample was enzymatically cut, desalted, centrifuged, and dried in a vacuum. The peptide segment was redissolved by 0.1% formic acid in water and then applied to the machine for chromatographic separation and mass spectrometry collection. BioPharma Finder5.1 software was used to analyze the glycosylation modification information of the samples, focusing on the glycosylation information at the Asn297 site. Data processing parameters: Protease (Trypsin/Chymotrypsin/Protease K); Variable Modification (Deamidation, Oxidation, Glycosylation); Fixed Modification (Carbamidomethylation); Mass Accuracy (20 ppm).

### 5.12 Anti‐hCLDN6 IgG1 Activity Analysis

The affinity of IgG1 was analyzed by detecting the signal of serially diluted antibodies binding to HEK293T‐hCLDN6 cells using flow cytometry. In a 96‐well assay plate, 30 µL of HEK293T‐hCLDN6 cell suspension and 30 µL of the corresponding diluted antibody were added to each well, followed by the Antibody‐Dependent Cell‐Mediated Cytotoxicity (ADCC) and Complement‐dependent cytotoxicity (CDC) assay. For the ADCC assay, 30 µL of Jurkat‐CD16A‐NFAT‐Luc cell suspension (E/T ratio = 2) was added to the pre‐incubated antibody‑HEK293T‐hCLDN6 mixture. After incubation at 37 °C for 6 h, 50 µL/ well of One‐Glo reagent was added to each well, and luminescence intensity was measured using a microplate reader (Tecan). For CDC assay, the freshly diluted human complement serum was added to the pre‐incubated antibody‑HEK293T‐hCLDN6 mixture. After incubation for 2 h, cell viability was detected using the CellTiter‐Glo assay to calculate the specific cell lysis percentage.

### 5.13 Statistical Analysis

All statistical analyses were performed using R software, GraphPad Prism, and SPSS. For two‐group comparisons, the unpaired two‐tailed Student's *t*‐test was used for normally distributed data, and the Wilcoxon rank‐sum test was used for non‐normal data. Dose‐response curves of ADCC and CDC activities were fitted by four‐parameter logistic (4PL) nonlinear regression in GraphPad Prism. The half‐maximal effective concentration (EC50) was calculated to evaluate antibody potency. Differences in dose‐response curves and EC50 between mAb01‐C and mAb01‐CS were compared by an extra sum‐of‐squares F‐test. Survival curves were generated using the Kaplan–Meier method, and inter‐group survival differences were compared using the two‐sided log‐rank test. Univariate and multivariate Cox proportional hazards regression models were used to screen independent prognostic factors. Hazard ratios (HRs) and 95% confidence intervals (CIs) were calculated for all clinical and pathological variables.

## Conflicts of Interest

The authors declare no Conflicts of interest.

## Supporting information




**Supporting File**: advs75790‐sup‐0001‐SuppMat.docx.

## Data Availability

The mass spectrometry proteomics data that support the findings of this study are openly available in PRIDE (PXD058527). The microbiome profiling data are available in BioSample (SUB15227361). The RNA‑seq data are available in the NCBI Sequence Read Archive (SRA) (PRJNA1223658). Additional data that support the findings of this study are available from the corresponding author upon reasonable request.
