# Supplementary material for: SSR4 sustains Tertiary Lymphoid Structures by Regulation Quality Control of N‐linked Glycosylation During B‐cell Differentiation Into Plasmacyte in Colorectal Cancer
Source: Adv Sci (Weinh). 2026 May 20:e75790. Online ahead of print. doi: 10.1002/advs.75790 (PMC13336112; doi:10.1002/advs.75790)
Supplement: Supplementary file 1 — Supporting File: advs75790‐sup‐0001‐SuppMat.docx. [file ADVS-9999-e75790-s001.docx]

**List of Supplementary Materials**

**KEY RESOURCES TABLE**

| REAENT or RESOURCE | SOURCE | IDENTIFIER | Reactivity |
| --- | --- | --- | --- |
| ANTIBODIES for IMMUNOHISTOCHEMISTRY |  |  |  |
| Rabbit polyclonal anti-SSR4 antibody | Proteintech | Cat No. 11655-2-AP RRID AB_2195738 | Human, Mouse, Rat |
| Rabbit monoclonal anti-Ki67 antibody (SP6) | Abcam | Cat No. ab21700 | Mouse, Human |
| Rabbit monoclonal anti-PAX5 antibody (1H9) | Abcam | Cat No. ab211293 | Mouse, Human |
| Rabbit monoclonal anti-CD10 antibody (SP67) | Abcam | Cat No. ab227640 | Human |
| Rabbit monoclonal antii-BCL-2 antibody (BCL2/1878R) | Abcam | Cat No. ab238042 | Human |
| Rabbit monoclonal anti-CD3 antibody (SP162) | Abcam | Cat No. ab135372 | Mouse, Human |
| Rabbit monoclonal anti-CD19 antibody (RM1166) | Abcam | Cat No. ab317335 | Mouse, Human |
| ANTIBODIES for IMMUNOFLUORESCENCE |  |  |  |
| Rabbit monoclonal anti-EPCAM antibody (EPR20532-222) | Abcam | Cat No. ab213500 | Human, Mouse, Rat |
| Rabbit polyclonal anti-SSR4 antibody | Proteintech | Cat No. 11655-2-AP RRID AB_2195738 | Human, Mouse, Rat |
| Rabbit polyclonal anti-DDOST antibody | Abcam | Cat No. ab204314 | Human, Mouse, Rat |
| Rabbit monoclonal anti-CAD79b antibody (EPR6861) | Abcam | Cat No. ab134147 | Mouse, Rat, Huma |
| Rabbit monoclona anti-CD4 antibody (EPR19514) | Abcam | Cat No. ab183685 | Mouse |
| Rabbit monoclona anti-CD8 antibody (EPR20305) | Abcam | Cat No. ab209775 | Mouse |
| Rabbit monoclonal anti-CD8 antibody | Abcam | Cat No.sp16 | Human |
| Rabbit monoclonal anti-CD4 antibody | Abcam | Cat No. EPR6855 | Human |
| Mouse Monoclonal anti-panCK antibody | ZSGB-BIO | Cat No. ZM-0069 | Human, Mouse |
| Rat monoclonal antibody anti-CD3 antibody | Abcam | Cat No. ab11089 | Human, Mouse, Rat |
| Rabbit Monoclonal anti-CD20 antibody | Abcam | Cat No. ab64088 | Human, Mouse, Rat |
| Rabbit monoclonal anti-Ki67 antibody (SP6) | Abcam | Cat No. ab21700 | Mouse, Human |
| Rabbit monoclonal anti-CD38 antibody | Abcam | Cat No. ab108403 | Human, Mouse |
| Rabbit monoclonal anti-CD19 antibody (EPR23174-145) | Abcam | Cat No. ab245235 | Mouse |
| ANTIBODIES for WESTERN BLOT |  |  |  |
| Rabbit monoclonal anti-CD19 antibody (EPR23174-145) | Abcam | Cat No. ab245235 | Mouse |
| Rabbit monoclonal anti-CD19 antibody (EPR5906) | Abcam | Cat No. ab134114 | Human |
| Rabbit polyclonal RPN1 antibody | Proteintech | Cat No. 12894-1-AP  RRID AB_2170474 | Human, Mouse, Rat |
| Rabbit polyclonal anti-DDOST antibody | Abcam | Cat No. ab204314 | Human, Mouse, Rat |
| Rabbit polyclonal anti-SSR4 antibody | Proteintech | Cat No. 11655-2-AP  RRID AB_2089878 | Human |
| Rabbit monoclonal anti-RelB antibody (D7D7W) | CST | Cat No. 10544 | Human, Mouse, Rat |
| Rabbit monoclonal anti-P100/P52 antibody | CST | Cat No. 4882T | Human, Mouse, Rat |
| Mouse monoclonal anti-GAPDH antibody (FF26A) | abcam | Cat No. ab59164 | human |
| Mouse monoclonal anti--actin antibosy (ab8226) | abcam | Cat No. ab8226 | Mouse, human,Rat |
| Rabbit polyclonal to HA tag | abcam | Cat No. Ab9110 |  |
| Mouse monoclonal to Flag tag | Proteintech | Cat No. 66008-4-AP |  |
| Goat polyclonal anti-Lamin B antibody (C-20) | SANTA CRUZ | Cat No. sc-6216 | Mouse, human,Rat |
| Mouse monoclonal anti-Tubulin antibody | LMAI Bio | Cat No. LM33034M | Mouse, human,Rat |
| Mouse monoclonal anti-RFP antibody (7A5) | Bioss | Cat No. Bsm-33011M | N/A |
| Mouse monoclonal anti-BAFF-R antibody (H-1) | SANTA CRUZ | Cat No. sc-365410 | Mouse, human,Rat |
| Rabbit Recombinant Monoclonal Lta antibody | Abcam | Cat No: AB314858 | Mouse |
| Rabbit polyclonal anti- LC3 antibody | proteintech | Cat No: Ag2781 | Mouse, human,Rat |
| Rabbit Polyclonal P-eIF2a antibody | Beyotime | Cat No: AF5803 | Mouse, human,Rat |
| Mouse monoclonal anti-eIF2a antibody | Beyotime | Cat No: AG1817 | Mouse, human,Rat |
| ANTIBODIES for FLOW CYTOMETRY |  |  |  |
| PerCP Rat Anti-Mouse B220/CD45R (RA3-6B2) antibody | BD Biosciences | Cat No. 561086 RRID AB_394622 | Mouse, Human |
| V450 Rat Anti-Mouse CD19 (1D3) antibody | BD Biosciences | Cat No. 560376 RRID AB_1645269 | Mouse |
| APC-antiCD21(SP199) antibody | Abcam | Cat No. ab306510 | Mouse |
| PE Rat Anti-Mouse CD23 (B3B4) antibody | BD Biosciences | Cat No. 553139 RRID AB_394654 | Mouse |
| PE Rat anti-Mouse CD43 (S7) antibody | BD Biosciences | Cat No. 560199 RRID AB_10926206 | Mouse |
| APC-anti IgM (145-8) antibody | BD Biosciences | Cat No.345160 | Mouse, Human |
| BV510 Rat Anti-Mose IgD (11-26c.2a) antibody | BD Biosciences | Cat No. 563110 RRID AB_2737003 | Mouse |
| Rat Anti-Mouse CD138 antibody | BD Biosciences | Cat No. 553712  RRID AB_394998 | Mouse |
| FITC anti-mouse/human GL7 antibody | BD Biosciences | Cat No. 144603  RRID AB_2561696 | Mouse, Human |
| PE-Cy7-anti-mouse-CD86 | BD Biosciences | Cat No.560582  RRID AB_1727518 | Mouse |
| FITC-anti-mouse-CD69 | BD Biosciences | Cat No. 561929  RRID AB_396675 | Mouse |
| APC-anti-mouse-MHC-II | Elabscience | Cat No. E-AB-F0990E | Mouse |
| CHEMICALS, PEPTIDES, and RECOMBINANT PROTEINS |  |  |  |
| DAPT | ABCAM | Cat No. ab120633 |  |
| NP-KLH | LGC Biosearch Technologies | Cat No. N-5060-1 |  |
| TNP-LPS | LGC Biosearch Technologies | Cat No. N-5065-1 |  |
| Recombinant Human BAFF | Thermo Fisher Scientific | Cat No. PHC1674 |  |
| LPS | Sigma-Aldrich | Cat No. L8654 |  |
| CFA | Sigma-Aldrich | Cat No. 08168 |  |
| IFA | MCE | Cat No. HY-153808 |  |
| Puromycin | Sigma-Aldrich | Cat No.P8833 |  |
| CFSE | Invitrogen^TM^ | Cat No.34554 |  |
| AffiniPure F(ab’)2 Fragment Goat anti-mouse IgM | Jackson ImmunoResearch | Cat No.115-006-020 |  |
| Recombinant murine IL-4 | Jackson ImmunoResearch | Cat No. 214-14 |  |
| CD40 | BioXCell | Cat No. BP0016 |  |
| CRITICAL COMMERCIAL ASSAYS |  |  |  |
| Pierce BCA Protein Assay Kit - Reducing Agent Compatible | Thermo Scientific | Cat No.23250 |  |
| Phospholipid Assay Kit (Colorimetric/ Fluorimetric) | Abcam | Cat No.ab234050 |  |
| RNeasy Mini Kit | Qiagen | Cat No.74104 |  |
| RNase-Free DNase Set | Qiagen | Cat No.79254 |  |
| RevertAid RT Reverse Transcription Kit | Thermo Scientific | Cat No.K1691 |  |
| SOLIScript RT cDNA | Solis BioDyne | Cat No.06-35-0000S |  |
| CD19 MicroBeads mouse | Miltenyi Biotec | Cat No. 130121301 |  |
| Cytoplasmic nuclear separation kit | invent | Cat No.SC-003 |  |
| ONE-Glo™ Luciferase Assay System | Promega | Cat No. E6120 |  |
| CellTiter-Glo® Luminescent Cell Viability Assay | Promega | Cat No. G7572 |  |
| DAPI | Merck | Cat No.D9542 |  |
| CELL LINES |  |  |  |
| DAUDI | YaJi | Cat No. YS461C |  |
| 293[HEK-293] | ATCC | Cat No. CRL-1573  RRID:CVCL_9804 |  |
| CHOS | Invitrogen | Cat No. AC-2203H |  |
| Jurkat-CD16A-NFAT-Luc | Genomeditech | Cat No. GM-C01459 |  |
| MC38-F-Luc-PURO | BIOWING | Cat No. 20251014-01 |  |
| Experimental models: Organisms/strains |  |  |  |
| Mouse: C57BL/6J | GemPharmatech | N/A |  |
| Mouse: *Ssr4* | GemPharmatech | N/A |  |
| Mouse: *Cd1*9-Cre | GemPharmatech | N/A |  |
| Mouse: *Apc*-min | GemPharmatech | N/A |  |
| Deposited data |  |  |  |
| Analysis data of spatial transcriptome of colorectal cancer samples | Human Intestine Cancer (FPPE) COLON MAP: Colon Molecular Atlas Project | TENX49  MISC35  MISC36  MISC65 |  |
| Processed single-cell RNA-seq data of  human CRC patients | Deeply Integrated human Single-Cell Omics database | GSM7290763  GSM7290769  GSM7290772  GSM7290773  GSM7290774  GSM7290777  GSM7349819 |  |
|  | Chen et al. (2024) | GEO: GSE236581 |  |
| The mass spectrometry proteomics data | This paper | PRIDE：PXD058527 |  |
| Microbiome profiling data | This paper | BioSample: SUB15227361 |  |
| RNA-seq data | This paper | NCBI(SRA)：PRJNA1223658 |  |
| Software and algorithms |  |  |  |
| FlowJo v10 | Tree star | https://www.flowjo.com/ |  |
| Prism v9.0 | Graphpad | https://www.graphpad.com/features |  |
| SPSS v23.0 | IBM | https://www.ibm.com/cn-zh/products/spss-statistics |  |
| GSEA v4.1.0 | Broad Institute | index.jsp |  |
| R v4.0.2 | Robert Gentleman and Ross Ihaka | https://www.r-project.org/ |  |
| Image J | Wayne Rasband | https://imagej.net/software/fiji/downloads |  |
| Qupath v0.6.0 | N/A | https://qupath.github.io/ |  |
| Seurat v4.1.1 | N/A | https://github.com/satijalab/seurat |  |
| BioPharma Finder5.1 | ThermoFisher | https://thermoffsher.com/BioPharmaFinder |  |
| Byonic | Protein Metrics | https://binocvision.cn/proteinmetrics/byonic/ |  |
| Monocle3 v1.2.9 | N/A | https://cole-trapnell-lab.github.io/monocle3/ |  |

**Supplementary Materials**

**Table S1 The information of 4 spatial transcriptome CRC tissue samples**

| **Dataset_title** | **ID** | **Organ** | **Oncotree_code** | **Species** | **License** | **Download_page_link1** |
| --- | --- | --- | --- | --- | --- | --- |
| Human Intestine Cancer (FPPE) | TENX49 | Bowel | COAD | Homo sapiens | Creative Commons Attribution | https://www.10xgenomics.com/datasets/human-intestine-cancer-1-standard |
| COLON MAP: Colon Molecular Atlas Project | MISC35 | Bowel | COAD | Homo sapiens | CC BY-NC-ND 4.0 | https://humantumoratlas.org/explore?selectedFilters=%5B%7B%22value%22%3A%22HTAN+Vanderbilt%22%2C%22group%22%3A%22AtlasName%22%2C%22count%22%3A2389%2C%22isSelected%22%3Afalse%7D%2C%7B%22value%22%3A%2210x+Visium%22%2C%22group%22%3A%22assayName%22%2C%22count%22%3A369%2C%22isSelected%22%3Afalse%7D%2C%7B%22value%22%3A%22hdf5%22%2C%22group%22%3A%22FileFormat%22%2C%22count%22%3A41%2C%22isSelected%22%3Afalse%7D%2C%7B%22value%22%3A%22tif%22%2C%22group%22%3A%22FileFormat%22%2C%22count%22%3A41%2C%22isSelected%22%3Afalse%7D%5D&tab=file |
| COLON MAP: Colon Molecular Atlas Project | MISC36 | Bowel | COAD | Homo sapiens | CC BY-NC-ND 4.0 | https://humantumoratlas.org/explore?selectedFilters=%5B%7B%22value%22%3A%22HTAN+Vanderbilt%22%2C%22group%22%3A%22AtlasName%22%2C%22count%22%3A2389%2C%22isSelected%22%3Afalse%7D%2C%7B%22value%22%3A%2210x+Visium%22%2C%22group%22%3A%22assayName%22%2C%22count%22%3A369%2C%22isSelected%22%3Afalse%7D%2C%7B%22value%22%3A%22hdf5%22%2C%22group%22%3A%22FileFormat%22%2C%22count%22%3A41%2C%22isSelected%22%3Afalse%7D%2C%7B%22value%22%3A%22tif%22%2C%22group%22%3A%22FileFormat%22%2C%22count%22%3A41%2C%22isSelected%22%3Afalse%7D%5D&tab=file |
| COLON MAP: Colon Molecular Atlas Project | MISC65 | Bowel | COAD | Homo sapiens | CC BY-NC-ND 4.0 | https://humantumoratlas.org/explore?selectedFilters=%5B%7B%22value%22%3A%22HTAN+Vanderbilt%22%2C%22group%22%3A%22AtlasName%22%2C%22count%22%3A2389%2C%22isSelected%22%3Afalse%7D%2C%7B%22value%22%3A%2210x+Visium%22%2C%22group%22%3A%22assayName%22%2C%22count%22%3A369%2C%22isSelected%22%3Afalse%7D%2C%7B%22value%22%3A%22hdf5%22%2C%22group%22%3A%22FileFormat%22%2C%22count%22%3A41%2C%22isSelected%22%3Afalse%7D%2C%7B%22value%22%3A%22tif%22%2C%22group%22%3A%22FileFormat%22%2C%22count%22%3A41%2C%22isSelected%22%3Afalse%7D%5D&tab=file |

**Table S2 Pathological and treatment information of 4 colorectal cancer samples from single-cell sequencing data**

| **Patient ID** | **Age** | **Gender** | **Cancer Type** | **Tumor Location** | **TNM** | **Tumor stage** | **Response** |
| --- | --- | --- | --- | --- | --- | --- | --- |
| P01 | 51 | Male | CRC | Descending colon | T4bN0M0 | II | CR |
| P08 | 52 | Male | CRC | Low rectum | T3N1M0 | III | CR |
| P09 | 64 | Male | CRC | Low rectum | T3bN2b | III | CR |
| P14 | 58 | Male | CRC | Low rectum | T3N2bM0 | III | PR |

**Table S3 Differentially expressed glycoproteins and glycosylation sites in BKO/WT**

| **Gene name** | **Localization probability** | **PEP** | **Score** | **Charge** | **Modified sequence** |
| --- | --- | --- | --- | --- | --- |
| Cd19 | 1 | 1.2542E-222 | 402.72 | 2 | HTLLEYLLEEGN(1)LSR |
| Ms4a1 | 1 | 2.5982E-193 | 284.14 | 3 | RLELIQTSKPYVDIYDCEPSN(1)SSEK |
| Ncstn | 1 | 9.70722E-72 | 210.5 | 3 | LLN(1)ATHQIGCQSSISGDTGVIHVVEKEEDLK |
| Tnfrsf13c | 1 | 1.47158E-20 | 162.16 | 3 | DSSVPTQCN(1)QTECFDPLVR |
| Pld4 | 1 | 2.70048E-66 | 203.93 | 4 | N(1)ISVVVATHSPTLAKTSTDLQVLAAHGAQIR |
| Tlr9 | 1 | 1.3803E-173 | 311.71 | 4 | GIGHN(1)FSFVTHLSMLQSLSLAHNDIHTR |
| Hsp90b1 | 1 | 6.50357E-93 | 238.33 | 2 | TDDEVVQREEEAIQLDGLN(1)ASQIR |
| Rpn1 | 1 | 2.4332E-171 | 278.01 | 4 | TILPAAAQDVYYRDEIGN(1)VSTSHLLILDDSVEMEIRPR |
| Lman2 | 0.96404 | 9.724E-38 | 195.36 | 3 | VFPYISVMVN(0.036)N(0.964)GSLSYDHSK |
| Stt3b | 1 | 0.00000483672 | 140.05 | 2 | AMSSN(1)ETAAYK |
| Itgal | 1 | 6.45817E-92 | 224.07 | 3 | VLVIITDGEASDKGN(1)ISAAHDITR |
| Btla | 1 | 1.29365E-16 | 171.34 | 3 | YCVHRPN(1)VTWCK |

**Table S4 Extracellular domain binding docking score**

| **Rank** | **Non Cytoplamic** | |
| --- | --- | --- |
|  | **Docking Score** | **Confidence Score** |
| 1 | -266.39 | 0.9217 |
| 2 | -252.1 | 0.8759 |
| 3 | -240.3 | 0.7696 |
| 4 | -238.28 | 0.7623 |
| 5 | -205.59 | 0.7525 |
| 6 | -203.16 | 0.7433 |
| 7 | -202.94 | 0.7425 |
| 8 | -202.26 | 0.7399 |
| 9 | -201.9 | 0.7385 |
| 10 | -201.56 | 0.7371 |

**Table S5 Primer for genotyping**

| **Primer Name** | **Sequence (5’-3’)** | **PCR size** | **Primer illustration** |
| --- | --- | --- | --- |
| JS05932-*Ssr4*-5wt-tF1 | AAGGATTCCAATTCACAGGGTGC | Wt=288bp  Fl=390bp | Identify SSR4 |
| JS05932-*Ssr4*-5wt-tR1 | AAGAGCAGGTTAAAGGTTCAGGC |  |  |
| JS05932-*Ssr4*-5wt-tF1 | AAGGATTCCAATTCACAGGGTGC | Null  431bp |  |
| JS05932-*Ssr4*-5wt-tR1 | CCACATAGGAGACATCACAAAGGC |  |  |
| YF000118-KI-tF1 | GCCTTTGAACGCACTGACTTTG | Ki= 227bp | Identidy Cd19-cre |
| YF000118-KI-tR1 | TTCTCCACACCAGCTGTGGACA |  |  |
| YF000118-wt-tF3 | TATGCAGCTCCTCAGCTCCACT | Ki=1630bp  Wt=517bp |  |
| YF000118-wt-tR3 | TTCACTGACTGACACCATCTGGG |  |  |

**Table S6 Clinical information of 10 patients with colorectal cancer**

|  | Pathology ID | Gender | Age | Morphological classification | Pathological cllassification | 8th pTNM stage |
| --- | --- | --- | --- | --- | --- | --- |
| 1 | 22-12137D | female | 82 | eminence-type | adenocarcinoma | pT4bN0 |
| 2 | 22-05977E | male | 68 | ulcer-type | adenocarcinoma | pT3N1b |
|  | 22-05977H |  |  |  |  |  |
| 3 | 22-06994F | male | 69 | eminence-type | adenocarcinoma | pT3N0 |
| 4 | 22-12071D | female | 76 | eminence-type | adenocarcinoma | pT3N1c |
| 5 | 22-09433C | female | 84 | eminence-type | adenocarcinoma | pT3N0 |
| 6 | 22-14303D | female | 87 | eminence-type | adenocarcinoma | pT3N0 |
| 7 | 22-09106D | female | 71 | ulcer-type | adenocarcinoma | pT3N0 |
| 8 | 22-05901I | male | 76 | ulcer-type | adenocarcinoma | pT3N0 |
| 9 | 22-09893E | female | 67 | ulcer-type | adenocarcinoma | pT3N1 |
| 10 | 22-13146B | female | 47 | eminence-type | adenocarcinoma | pT3N0 |

**Table S7 Clinical information of 120 patients with colorectal cancer**

| **Patient ID** | **Age** | **Gender** | **Morphological classification** | **Pathological classification** | **8th pTNM stage** |
| --- | --- | --- | --- | --- | --- |
| P01 | 62 | female | eminence-type | adenocarcinoma | pT3N0M0 |
| P02 | 69 | female | infiltration-type | adenocarcinoma | pT4N0M0 |
| P03 | 68 | female | eminence-type | adenocarcinoma | T3N0M0 |
| P04 | 70 | female | ulcer-type | adenocarcinoma | pT4N0M0 |
| P05 | 63 | male | ulcer-type | mucinous carcinoma | pT3N0M0 |
| P06 | 70 | female | eminence-type | mucinous carcinoma | T3N2M0 |
| P07 | 86 | male | eminence-type | adenocarcinoma | pT3N0M0 |
| P08 | 69 | male | ulcer-type | adenocarcinoma | pT3N0M0 |
| P09 | 64 | male | ulcer-type | adenocarcinoma | pT3N0M0 |
| P10 | 79 | female | ulcer-type | adenocarcinoma | pT2N0M0 |
| P11 | 63 | male | ulcer-type | adenocarcinoma | pT3N0M0 |
| P12 | 47 | female | ulcer-type | adenocarcinoma | pT4bN1a |
| P13 | 79 | male | ulcer-type | adenocarcinoma | pT3N1M0 |
| P14 | 70 | male | eminence-type | adenocarcinoma | pT3N0M0 |
| P15 | 61 | female | ulcer-type | adenocarcinoma | pT2N0M0 |
| P16 | 69 | female | eminence-type | adenocarcinoma | pT3N1M0 |
| P17 | 73 | female | eminence-type | adenocarcinoma | pT3N0M0 |
| P18 | 84 | female | eminence-type | adenocarcinoma | pT3N0M0 |
| P19 | 75 | female | ulcer-type | adenocarcinoma | pT3N0M0 |
| P20 | 81 | male | ulcer-type | adenocarcinoma | pT4aN1M0 |
| P21 | 60 | male | eminence-type | adenocarcinoma | pT3N0M0 |
| P22 | 60 | male | eminence-type | adenocarcinoma | pT3N1M0 |
| P23 | 77 | male | eminence-type | adenocarcinoma | pT2N1M0 |
| P24 | 60 | male | eminence-type | adenocarcinoma | pT3N1cM0 |
| P25 | 61 | female | eminence-type | adenocarcinoma | pT1N0M0 |
| P26 | 61 | female | eminence-type | adenocarcinoma | pT1N0M0 |
| P27 | 55 | male | ulcer-type | adenocarcinoma | pT3N0M0 |
| P28 | 51 | male | ulcer-type | adenocarcinoma | pT3N0M0 |
| P29 | 60 | male | ulcer-type | adenocarcinoma | pT31N1M0 |
| P30 | 82 | female | eminence-type | adenocarcinoma | pT3N1M0 |
| P31 | 54 | male | eminence-type | adenocarcinoma | pT3N0M0 |
| P32 | 77 | female | eminence-type | adenocarcinoma | pT4N0M0 |
| P33 | 52 | female | eminence-type | adenocarcinoma | pT3N1M0 |
| P34 | 64 | female | eminence-type | adenocarcinoma | pT3N1M0 |
| P35 | 63 | male | eminence-type | adenocarcinoma | pT3N0M0 |
| P36 | 82 | female | eminence-type | adenocarcinoma | pT3N0M0 |
| P37 | 89 | male | ulcer-type | adenocarcinoma | pT3N1M0 |
| P38 | 61 | male | ulcer-type | adenocarcinoma | pT3N0M0 |
| P39 | 80 | female | eminence-type | adenocarcinoma | pT3N1M0 |
| P40 | 72 | female | infiltration-type | adenocarcinoma | pT3N1M0 |
| P41 | 70 | female | ulcer-type | adenocarcinoma | pT3N2M0 |
| P42 | 69 | female | ulcer-type | adenocarcinoma | pT3N2M0 |
| P43 | 81 | male | eminence-type | adenocarcinoma | pT3N0M0 |
| P44 | 82 | male | eminence-type | adenocarcinoma | pT3N0M0 |
| P45 | 85 | male | ulcer-type | adenocarcinoma | pT3N1M0 |
| P46 | 74 | female | ulcer-type | adenocarcinoma | pT3N1M0 |
| P47 | 62 | male | eminence-type | adenocarcinoma | pT3N0M0 |
| P48 | 61 | male | eminence-type | adenocarcinoma | pT3N0M0 |
| P49 | 70 | male | ulcer-type | adenocarcinoma | pT3N0M0 |
| P50 | 69 | male | eminence-type | adenocarcinoma | pT3N0M0 |
| P51 | 73 | male | ulcer-type | adenocarcinoma | T3N0M0 |
| P52 | 64 | male | eminence-type | adenocarcinoma | 0T3N2M0 |
| P53 | 65 | male | eminence-type | adenocarcinoma | pT3N0M0 |
| P54 | 65 | male | eminence-type | adenocarcinoma | pT3N0M0 |
| P55 | 51 | male | eminence-type | adenocarcinoma | pT3N1M0 |
| P56 | 52 | male | eminence-type | adenocarcinoma | pT3N0M0 |
| P57 | 65 | female | eminence-type | adenocarcinoma | pT4aN0M0 |
| P58 | 60 | female | ulcer-type | adenocarcinoma | pT3N0M0 |
| P59 | 68 | female | infiltration-type | adenocarcinoma | pT3N0M0 |
| P60 | 69 | female | eminence-type | adenocarcinoma | pT2N0M0 |
| P61 | 80 | female | eminence-type | adenocarcinoma | pT3N1M0 |
| P62 | 81 | female | eminence-type | adenocarcinoma | pT3N0M0 |
| P63 | 80 | female | eminence-type | adenocarcinoma | pT3N1M0 |
| P64 | 56 | female | eminence-type | adenocarcinoma | pT3N1M0 |
| P65 | 56 | female | eminence-type | adenocarcinoma | pT3N0M0 |
| P66 | 61 | male | infiltration-type | mucinous carcinoma | pT4N0M0 |
| P67 | 68 | male | eminence-type | adenocarcinoma | pT3N1aM0 |
| P68 | 57 | male | eminence-type | adenocarcinoma | pT3N2M0 |
| P69 | 63 | female | ulcer-type | adenocarcinoma | pT3N1bM0 |
| P70 | 70 | male | eminence-type | adenocarcinoma | pT1N0M0 |
| P71 | 70 | female | eminence-type | adenocarcinoma | pT3N2M0 |
| P72 | 74 | female | ulcer-type | mucinous carcinoma | pT3N2bM0 |
| P73 | 73 | female | ulcer-type | mucinous carcinoma | pT3N0M0 |
| P74 | 81 | female | eminence-type | adenocarcinoma | pT2N0M0 |
| P75 | 78 | male | ulcer-type | mucinous carcinoma | pT3N1M0 |
| P76 | 88 | female | eminence-type | adenocarcinoma | pT1N1M0 |
| P77 | 76 | female | ulcer-type | mucinous carcinoma | pT3N0M0 |
| P78 | 76 | female | ulcer-type | mucinous carcinoma | pT3N0M0 |
| P79 | 63 | female | ulcer-type | mucinous carcinoma | pT4bN1aM0 |
| P80 | 64 | female | ulcer-type | mucinous carcinoma | pT4bN1aM0 |
| P81 | 68 | female | eminence-type | mucinous carcinoma | pT4aN2bM0 |
| P82 | 68 | female | eminence-type | mucinous carcinoma | pT4aN0M0 |
| P83 | 42 | female | eminence-type | adenocarcinoma | pT3N0M0 |
| P84 | 41 | female | eminence-type | adenocarcinoma | pT3N0M0 |
| P85 | 72 | male | eminence-type | adenocarcinoma | pT3N0M0 |
| P86 | 72 | male | eminence-type | adenocarcinoma | pT3N0M0 |
| P87 | 78 | male | ulcer-type | adenocarcinoma | pT3N1aM0 |
| P88 | 77 | male | ulcer-type | adenocarcinoma | pT3N0M0 |
| P89 | 64 | male | eminence-type | adenocarcinoma | pT2N0M0 |
| P90 | 65 | male | eminence-type | adenocarcinoma | pT2N0M0 |
| P91 | 73 | male | eminence-type | mucinous carcinoma | pT3N2bM0 |
| P92 | 73 | male | eminence-type | mucinous carcinoma | pT3N2bM0 |
| P93 | 59 | female | ulcer-type | adenocarcinoma | pT3N1aM0 |
| P94 | 59 | female | ulcer-type | adenocarcinoma | pT3N2bM0 |
| P95 | 57 | male | eminence-type | adenocarcinoma | pT1N1M0 |
| P96 | 57 | male | eminence-type | adenocarcinoma | pT3N1M0 |
| P97 | 78 | male | infiltration-type | adenocarcinoma | pT1N1M0 |
| P98 | 64 | female | ulcer-type | adenocarcinoma | pT3N0M0 |
| P99 | 64 | female | ulcer-type | adenocarcinoma | pT3N0M0 |
| P100 | 60 | male | ulcer-type | adenocarcinoma | pT3N0M0 |
| P101 | 68 | male | ulcer-type | adenocarcinoma | pT4N0M0 |
| P102 | 63 | male | eminence-type | mucinous carcinoma | pT3N0M0 |
| P103 | 72 | male | eminence-type | adenocarcinoma | pT3N0M0 |
| P104 | 72 | male | eminence-type | adenocarcinoma | pT3N0M0 |
| P105 | 80 | male | eminence-type | adenocarcinoma | pT3N0M0 |
| P106 | 68 | male | eminence-type | mucinous carcinoma | pT3N1M0 |
| P107 | 70 | male | eminence-type | mucinous carcinoma | pT3N0M0 |
| P108 | 69 | female | ulcer-type | adenocarcinoma | pT4aN2bM0 |
| P109 | 68 | female | ulcer-type | adenocarcinoma | pT4aN2bM0 |
| P110 | 61 | male | eminence-type | adenocarcinoma | pT3N0M0 |
| P111 | 72 | male | eminence-type | adenocarcinoma | PT3N0M0 |
| P112 | 61 | male | eminence-type | adenocarcinoma | pT3N0M0 |
| P113 | 60 | male | eminence-type | adenocarcinoma | pT3N1aM0 |
| P114 | 43 | male | infiltration-type | adenocarcinoma | pT3N0M0 |
| P115 | 42 | male | infiltration-type | adenocarcinoma | pT3N1aM0 |
| P116 | 45 | male | eminence-type | adenocarcinoma | pT1N0M0 |
| P117 | 35 | male | eminence-type | adenocarcinoma | pT3N0M0 |
| P118 | 21 | male | eminence-type | adenocarcinoma | pT3N0M0 |
| P119 | 61 | male | ulcer-type | adenocarcinoma | pT3N0M0 |
| P120 | 70 | male | ulcer-type | adenocarcinoma | pT3N0M0 |

**Table S8 Primer for plasmid and lentivirus**

| **Name** | **location** | **Primer** |
| --- | --- | --- |
| SSR4 (3FLG) | F1 | GTGGATCCGAGCTCGGTACCCGCCACCATGGCGGCGATGGCATCTCTC |
|  | R1 | ATATTTTATTACCGGTTTAATTAATCATTTGTCGTCATCATCCTTATAG |
| SR4 (del 24-144AA) -3FLG) | F1 | GTGGATCCGAGCTCGGTACCCGCCACCATGGCGGCGATGGCATCTCTC |
|  | R1 | ATATTTTATTACCGGTTTAATTAATCATTTGTCGTCATCATCCTTATAG |
| SSR4 (del 145-165AA) -3FLG) | F1 | GTGGATCCGAGCTCGGTACCCGCCACCATGGCGGCGATGGCATCTCTC |
|  | R1 | ATATTTTATTACCGGTTTAATTAATCATTTGTCGTCATCATCCTTATAG |
| SSR4 (del 166-173AA) -3FLG) | F1 | GTGGATCCGAGCTCGGTACCCGCCACCATGGCGGCGATGGCATCTCTC |
|  | R1 | ATATTTTATTACCGGTTTAATTAATCATTTGTCGTCATCATCCTTATAG |
| SSR4 (del E28/Q30/D124/T133/R139) | F1 | TACCGGACTCAGATCTCGAGCGCCACCATGGCGGCGATGGCATCTCTC |
|  | R1 | TCCTTGTAGTCCATGGATCCGGCCTGGATGTGGCTCTTCGCAC |
| SSR4 (del E28) | F1 | TACCGGACTCAGATCTCGAGCGCCACCATGGCGGCGATGGCATCTCTC |
|  | R1 | TCCTTGTAGTCCATGGATCCGGCCTGGATGTGGCTCTTCGCAC |
| SSR4 (del Q30) | F1 | TACCGGACTCAGATCTCGAGCGCCACCATGGCGGCGATGGCATCTCTC |
|  | R1 | TCCTTGTAGTCCATGGATCCGGCCTGGATGTGGCTCTTCGCAC |
| SSR4 (del D124) | F1 | TACCGGACTCAGATCTCGAGCGCCACCATGGCGGCGATGGCATCTCTC |
|  | R1 | TCCTTGTAGTCCATGGATCCGGCCTGGATGTGGCTCTTCGCAC |
| SSR4 (del T133) | F1 | TACCGGACTCAGATCTCGAGCGCCACCATGGCGGCGATGGCATCTCTC |
|  | R1 | TCCTTGTAGTCCATGGATCCGGCCTGGATGTGGCTCTTCGCAC |
| SSR4 (del R139) | F1 | TACCGGACTCAGATCTCGAGCGCCACCATGGCGGCGATGGCATCTCTC |
|  | R1 | TCCTTGTAGTCCATGGATCCGGCCTGGATGTGGCTCTTCGCAC |
| SSR4-RNAi (115964-1) |  | CAGGCACCTATGAGGTTAGAT |
| SSR4-RNAi (115965-11) |  | CAGAGGAATAACGAGGACATT |
| SSR4-RNAi (115966-1) |  | GTCCAGAACATGGCTCTCTAT |
| DDOST (3HA) | F1 | GTGGATCCGAGCTCGGTACCCGCCACCATGGAGCCCAGCACCGCGGC |
|  | R1 | AAAGATATTTTATTACCGGTTCATCAGGCGTAGTCAGGCACGTCATAAGGGTAAGCATAG |
| DDOST (del K327/G326/S295/R290/D104)-3HA) | F1 | GTGGATCCGAGCTCGGTACCCGCCACCATGGAGCCCAGCACCGCGGC |
|  | R1 | AAAGATATTTTATTACCGGTTCAGGCGTAGTCAGGCACGTCATAAGGGTAAGCATAG |

**Table S9 sgRNA and Primer sequence**

| Target | Seq Name | Target Seq（5’-3’） |
| --- | --- | --- |
| SSR4 | SSR4KO-sg1 | GATGGCATCTCTCGGCGCCC |
|  | SSR4KO-sg1-F1 | CTTACGCGTCGCTCTTCCTC |
|  | SSR4KO-sg1-R1 | CGGGGACTCCTCTTCGTATG |
|  | SSR4KO-sg2 | AGCGTCAGAAGTGGTGTAGT |
|  | SSR4KO-sg3 | CACAATGAAGACGGTCTCAG |
|  | SSR4KO-sg2/3-F1 | CCTTCCTTCACAGCCATCCC |
|  | SSR4KO-sg2/3-R1 | TTCTCCCGGTCCAGATACTC |
|  | SSR4KO-sg1 | GATGGCATCTCTCGGCGCCC |

**
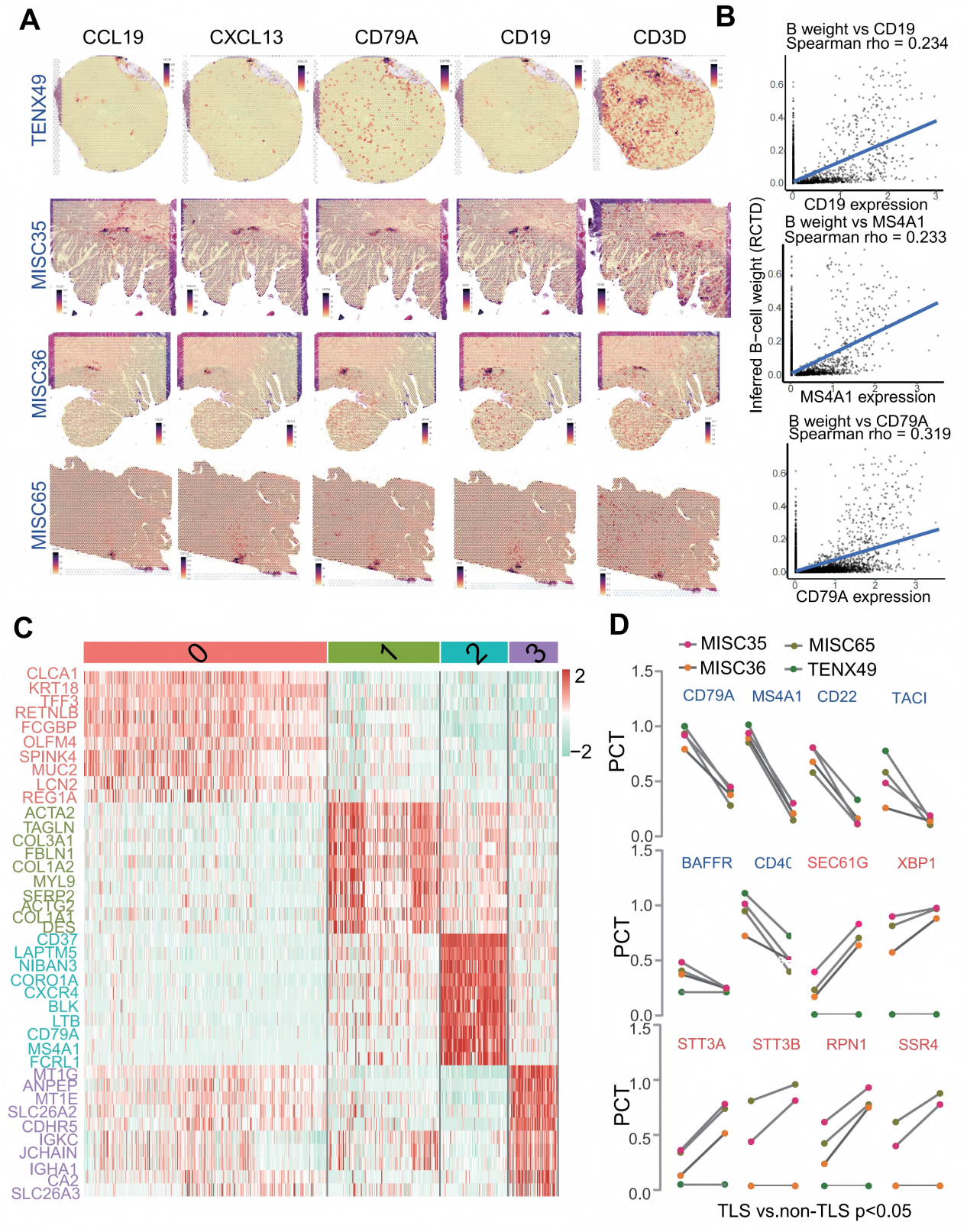
**

**Figure S1 Spatial transcriptomic profiling of B cells within tertiary lymphoid structures (TLS) in colorectal cancer (CRC)**

(**A**) The spatial expression information of the TLS marker genes, CCL19, CXCL13, CD79A, CD19 and CD3D, in 4 CRC tissue samples. (**B**) Scatter plot showing the positive correlation between B cell weight (Robust Cell Type Decomposition, RCTD) and expression levels of B cell surface markers. Correlation was assessed using Spearman’s rank correlation test (two-sided).

**
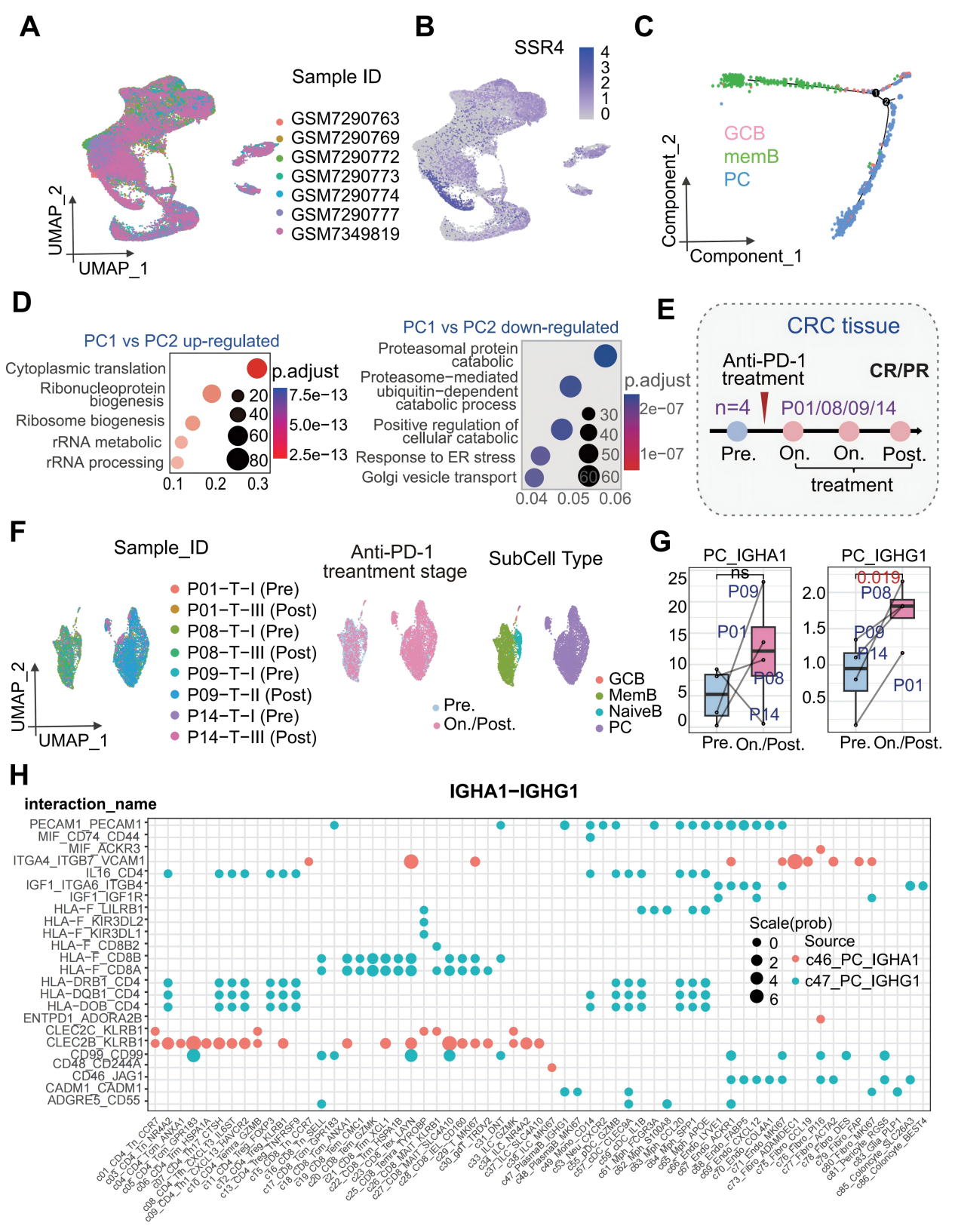
**

**Figure S2 Distribution of SSR4 expression in human colorectal cancer (CRC)**

(**A**) Uniform manifold approximation and projection (UMAP) plots visualization of 7 CRC tissue samples. (**B**) UMAP analysis of SSR4 mRNA expressed in 41,667 cells from single-cell data, n = 7. (**C**) Component plot showing the distinct differentiation direction and lineage progression of B cell subtypes, n = 7. (**D**) Dot plot showing the GO analysis results of the two types of PC cells; data are presented as dot plots; statistical significance was defined as adjusted *p* < 0.05. (**E**) Schematic diagram showing the sampled timepoints (GSE236581). (**F**) UMAP showing the treatment stage and B-cell subtypes and in 8 tissue samples of 4 additional CRC patients with paired pre- and post-PD-1 inhibitor treatment (GSE236581). (**G**) The box diagram shows the expression changes of SSR4 in IgHA1- and IgHG1- type PC at different treatment stages. Data are presented as box plots (n = 4 CRC patients, with paired pre- and post-PD-1 treatment samples); statistical significance was defined as adjusted *p* < 0.05. (**H**) The bubble chart shows the interactions between IGHA1-PC and IGHG1-PC and other types of cells. Cell-cell interaction analysis was performed using CellChat; data are presented as bubble charts.

**
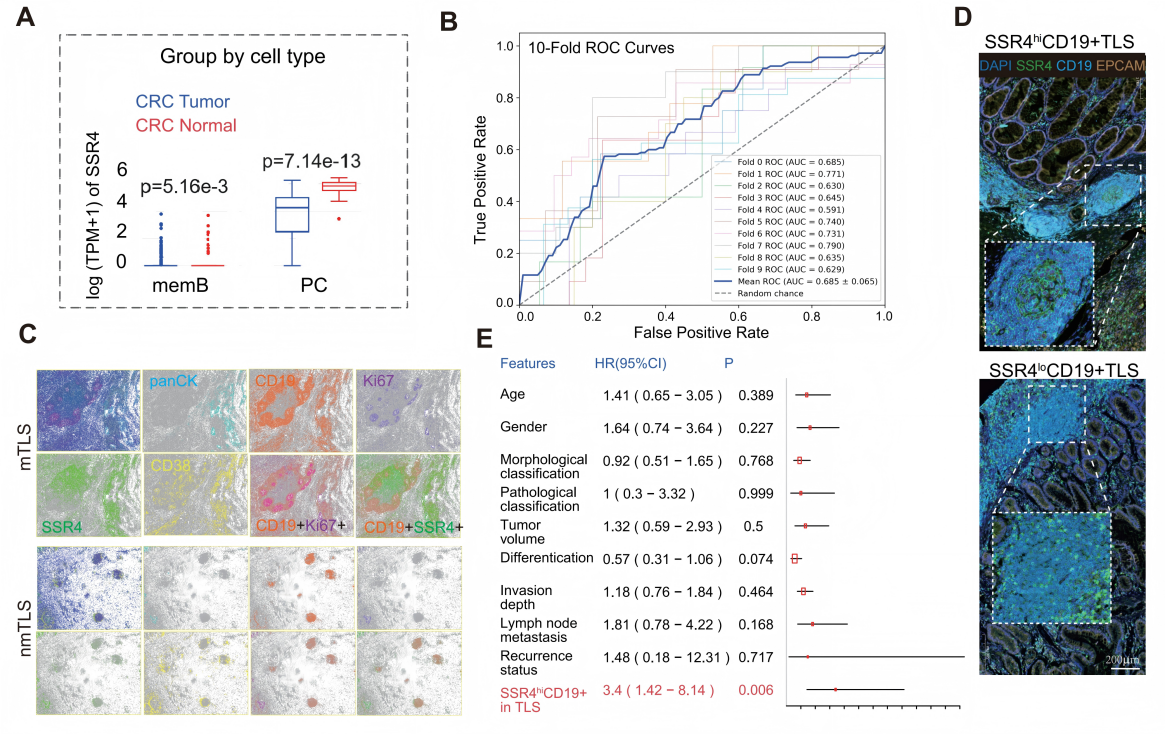
**

**Figure S3 Clinical sample analysis of B-cell subtypes and tertiary lymphoid structure (TLS) on the prognosis of colorectal cancer (CRC).**

(**A**) Analysis of the GEPIA databases revealed that SSR4 expression in memory B (memB) cells, and plasma cells (PC) was significantly higher in normal tissues than in CRC tissues. statistical significance was defined as *p* < 0.05. (**B**) The ten-fold cross-validation achieved a mean AUC of 0.685 ± 0.065, indicating stable predictive performance. Model performance was assessed using 10-fold cross-validation; data are presented as mean ± SD (AUC); sample size n = 299 slides (WSI-based AI model). (**C**) Representative images of integrated quantitative pathology analyses the spatial colocalization patterns of panCK, CD19, SSR4, CD38, Ki67 within TLS from human CRC tissues (sample size n = 11 tissue samples from 10 CRC patients). Representative images are shown (**D**) Representative mIF images of CD19 and SSR4 within TLS from human CRC tissues. Representative mIF images are shown. (**E**) The forest map shows that SSR^hi^CD19^+^ TLS is an independent prognostic factor for CRC patients. Survival analysis was performed using the Cox proportional hazards regression model; data are presented as forest plots (n = 120 TLS-positive CRC patients); statistical significance was defined as *p* < 0.05.

**
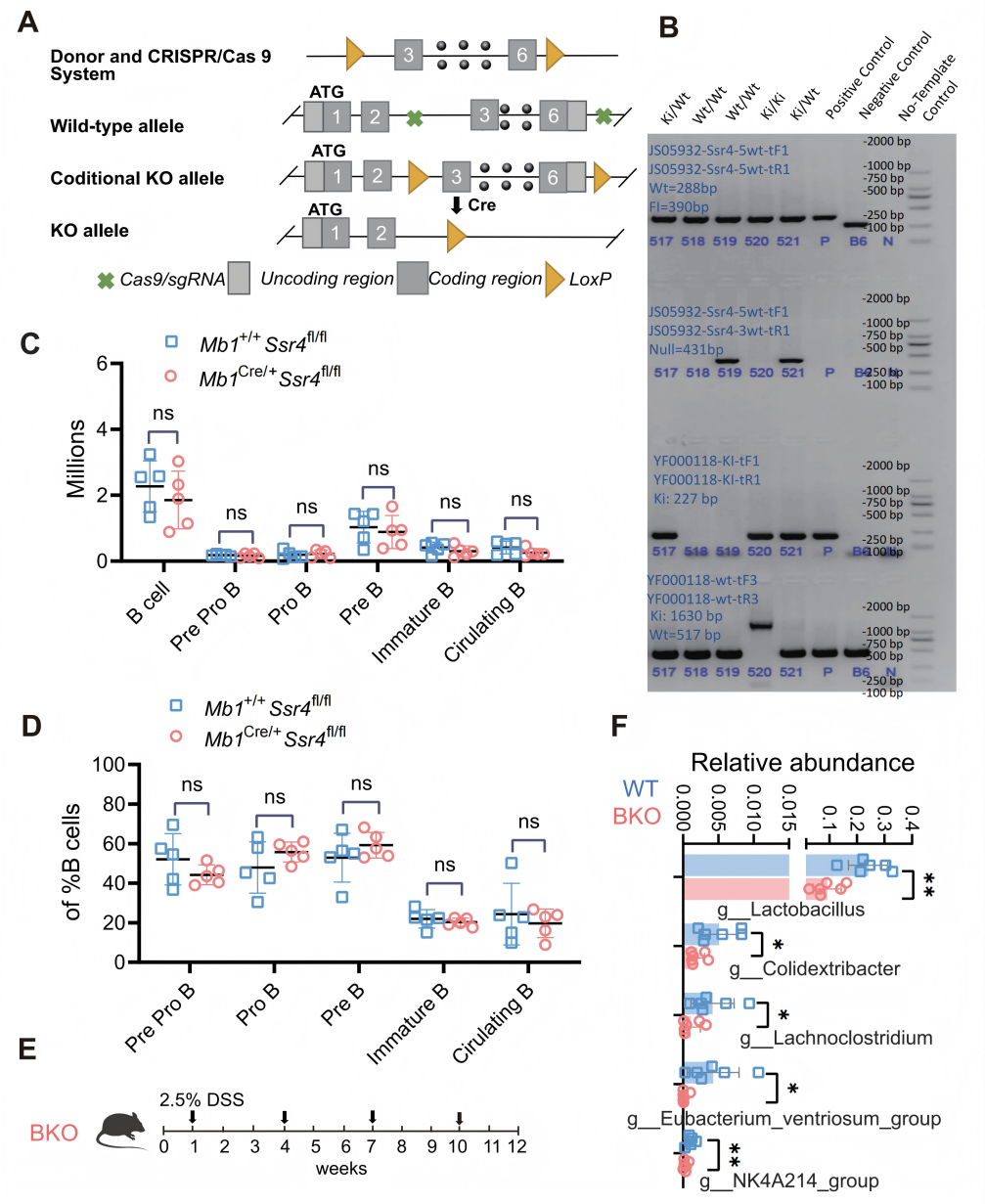
**

**Figure S4 Construction strategy and validation of conditional knockout mice and effects of SSR4 deletion on B-cell lineage development**

(**A**) Generation of B cell-specific *Ssr4* conditional knockout mice by crossing *Ssr4*^fl/fl^ mice with *Cd19*-Cre drivers. (**B**) Verification of gene levels of the constructed mouse model. (**C-D**) Flow cytometry analyses of the Pre Pro B cell, Pro B cell, Pre B cell, Immature b cell (IMB) and recirculation B cell in the bone marrow from adult *Ssr4*^fl/fl^;*Mb1*-Cre^ki/wt^ and *Ssr4*^fl/fl^;*Cd19*-Cre^wt/wt^ mice (8-10 weeks old). Group comparisons were performed using the two-tailed Student’s t-test; data are presented as representative plots and summary graphs; sample size n = 5 mice per group; statistical significance was defined as *p* < 0.05. (**E**) Schematic diagram of chronic enteritis model. (**F**) Differential expression of species abundance of commensal microbiota in the fecal extracts of age-matched *Ssr4*^BKO^ (BKO) and wild-type (WT) mice using 16S rRNA sequencing strategies. Group comparisons were performed using the Wilcoxon rank-sum test; each circle represents a mouse. n = 6 mice per group, **p* < 0.05, ***p* < 0.01.


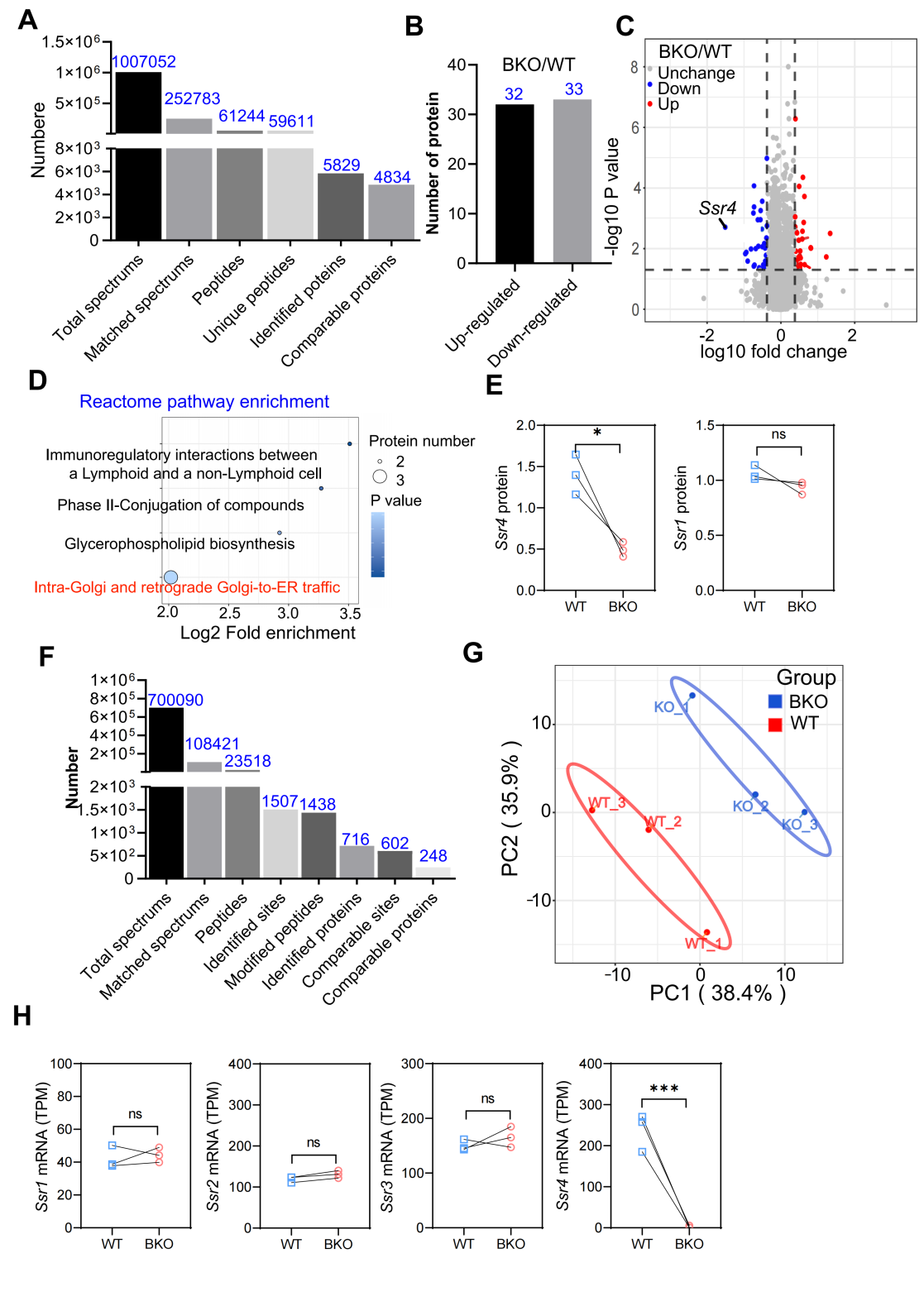


**Figure S5 N-linked glycosylation modification of** ***Ssr4* deletion in B cells**

(**A**) Overview of protein identification; data are presented as descriptive statistics; experiments were independently repeated three times. (**B**) The bar chart shows the number of up- and down- regulated protein. (**C**) The volcano plot shows the differential gene expression after the deletion of *Ssr4*. Differential expression analysis was performed using the two-sided Wilcoxon rank-sum test; data are presented as volcano plots; sample size n = 3 per group; statistical significance was defined as adjusted *p* < 0.05. (**D**) The bubble diagram shows enriched Reactome athway; data are presented as bubble charts; statistical significance was defined as adjusted *p* < 0.05. (**E**) The effect of *Ssr4* knockout on the protein expression levels of the TRAP family. Each dot represents an individual biological replicate (n = 3). Group comparisons were performed using a two-tailed Student’s t-test. (**p* < 0.05); (**F**) Overview of modification site identification; data are presented as descriptive statistics; experiments were independently repeated three times. (**G**) Principal component analysis (PCA) of N-linked glycosylation modification omics. Data are presented as PCA plots; sample size n = 3 biological replicates. (**H**) The bar chart shows the changes in the mRNA levels of the TRAP family in spleen B cells induced by BAFF after the deletion of *Ssr4* in B cell. Each dot represents an independent biological replicate (n = 3). Statistical significance was determined using a two-tailed unpaired Student’s t-test (****p* < 0.001).

**
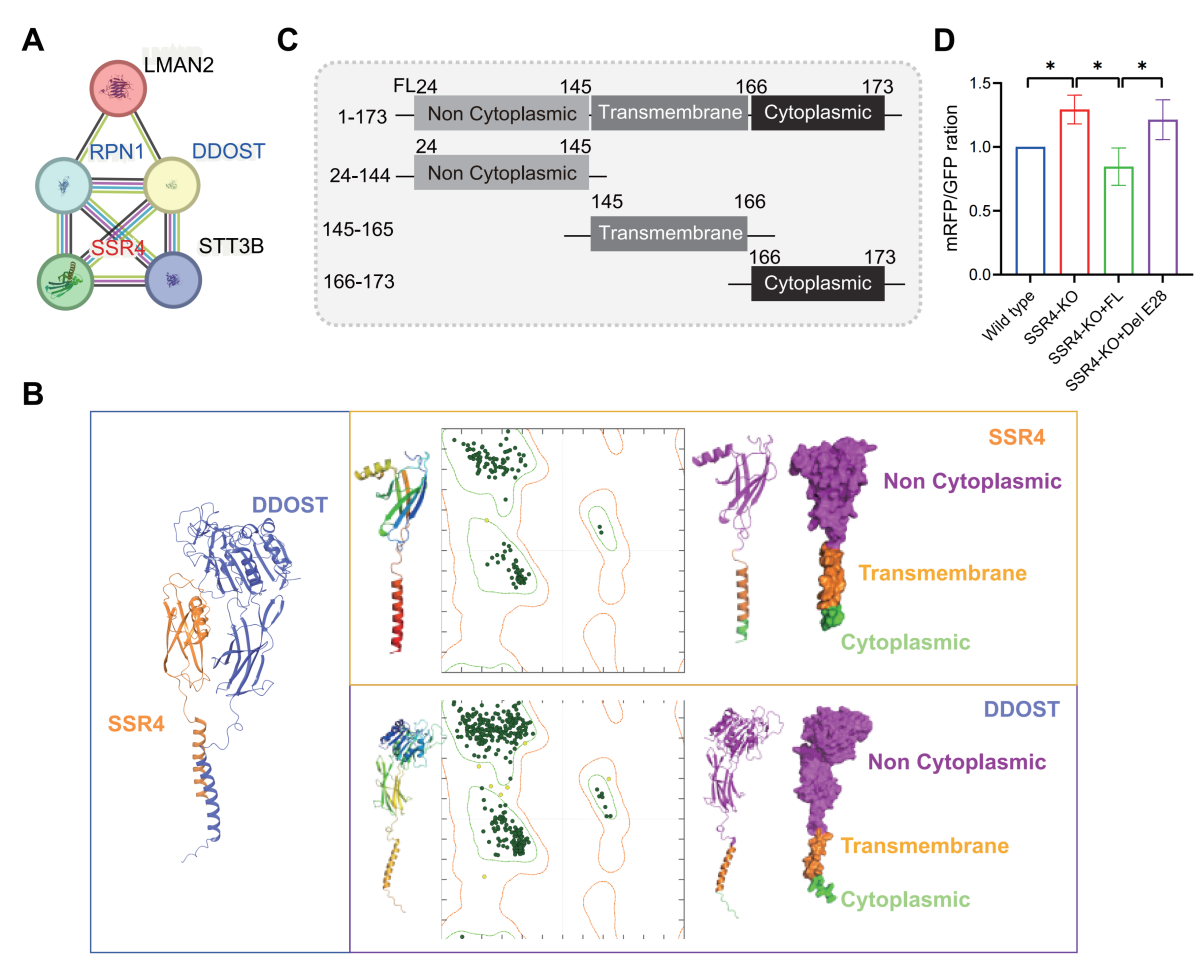
**

**Figure S6 Binding interaction of SSR4 and OST families**

(**A**) The STRING interaction network predicted an association between SSR4 and DDOST, a critical subunit of the oligosaccharyltransferase (OST) complex required for N-glycosylation quality control. (**B**) Alphafold2 predicts the structure of SSR4 and DDOST. (**C**) The structural compositions of SSR4. (**D**) The statistic graph of ER-phage in Daudi-KO cells. Group comparisons were performed using the unpaired two-tailed Student’s t-test; data are presented as mean ± SD (mRFP/mRFP-GFP ratio) (n = 3, **p* < 0.05).


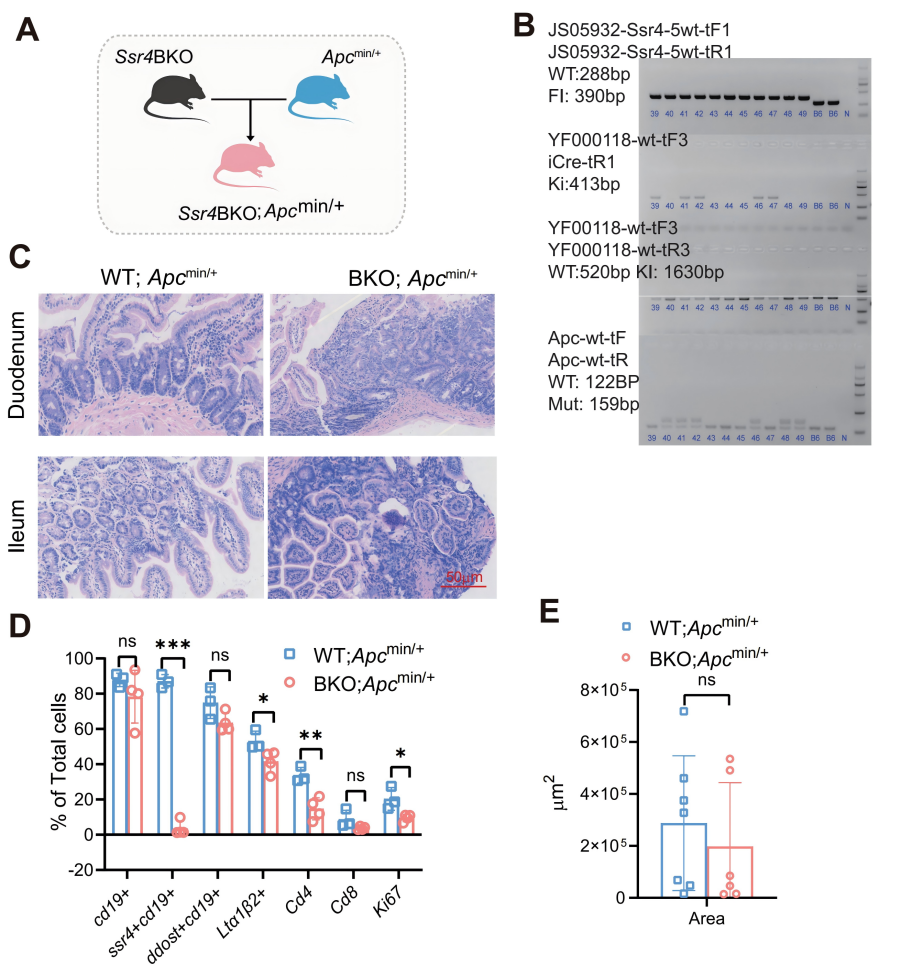


**Figure S7 Construction of the BKO;*Apc*^min/+^mice model and changes in TLS spatial distribution**

(**A**) Schematic diagram of BKO;*Apc*^min/+^mice model. (**B**) Verification of gene levels of the constructed mouse model. (**C**) Representative H＆E staining of the colorectal tissues from different groups. (**D**) The bar chart shows the proportion of immune cell subtypes in the two groups of TLS. Group comparisons were performed using the unpaired two-tailed Student’s t-test; data are presented as mean ± SD (cell proportion); sample size n = 6 mice per group (**p* < 0.05, ***p* < 0.01, ****p* < 0.001). (**E**) The quantitative analysis of the area of individual lymphocyte aggregates in the two groups. Group comparisons were performed using the two-tailed Student’s t-test; data are presented as mean ± SD).


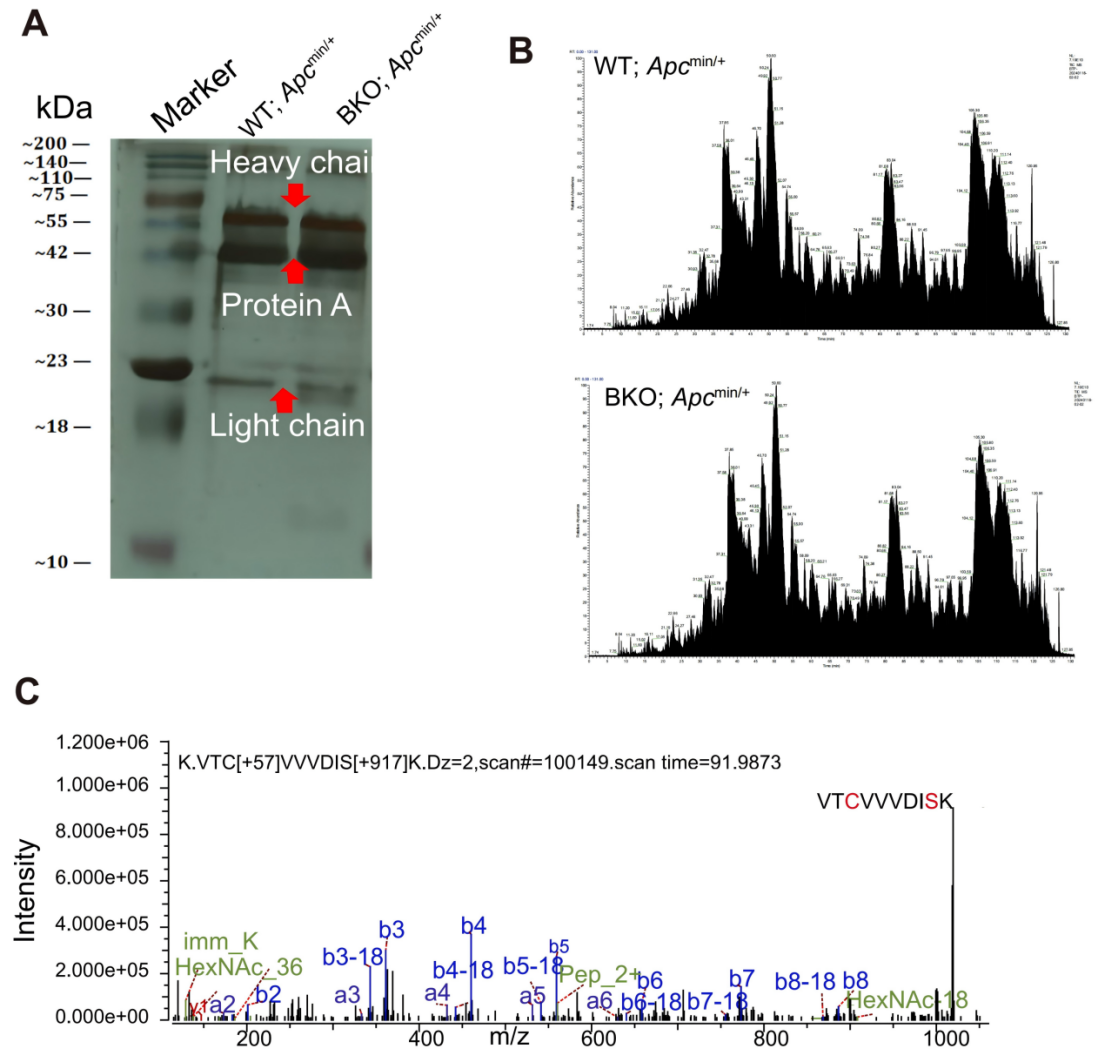


**Figure S8 Co-IP enrichment and mass spectrometric analysis of glycosylated serum IgG peptides in two-different types of mice models**

(**A**) Co-inmmunoprecipitation (Co-IP) assays on samples to enrich immunology (Ig). (**B**) the total ion chromatogram of the WT;*Apc*^min/+^ and BKO;*Apc*^min/+^ mice model serum sample, n = 3. (**C**) Mass spectrometry graph of the glycosylated peptide type of IgG1 in the serum samples of WT;*Apc*^min/+^ mice model.


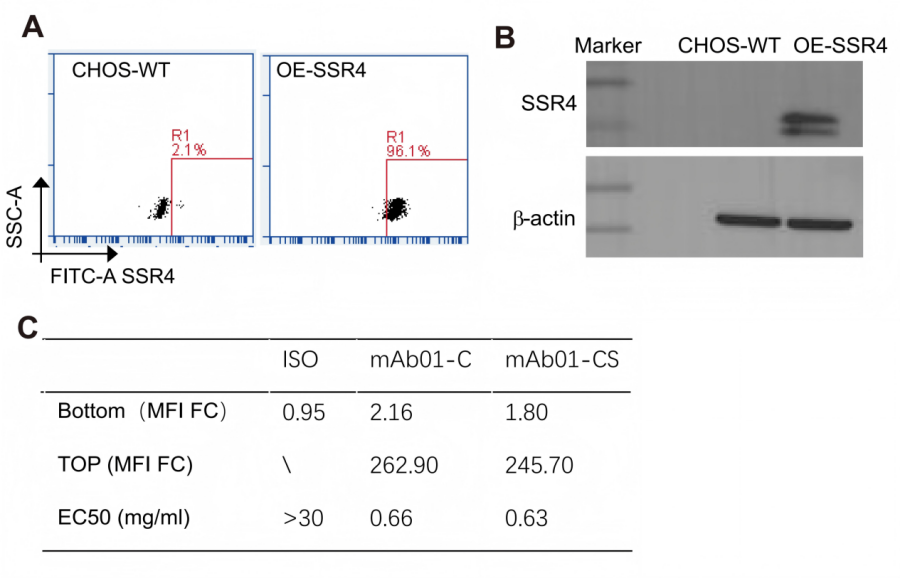


**Figure S9 Detection of CHOS-SSR4 Infection Efficiency**

Flow cytometry (**A**) and western blot (**B**) analyses CHOS-SSR4 Infection Efficiency. Data are presented as representative flow cytometry plots and western blot images. (**C**) Combining EC50 and Top median fluorescence intensity (MFI) fold change (FC).
